# Modeling the Cost-Effectiveness of Interventions to Prevent Plague in Madagascar

**DOI:** 10.3390/tropicalmed6020101

**Published:** 2021-06-11

**Authors:** Giovanni S. P. Malloy, Margaret L. Brandeau, Jeremy D. Goldhaber-Fiebert

**Affiliations:** 1 Department of Management Science and Engineering, Stanford University, Stanford, CA 94305, USA; brandeau@stanford.edu; 2 Stanford Health Policy, Centers for Health Policy and Primary Care and Outcomes Research, Stanford University, Stanford, CA 94305, USA; jeremygf@stanford.edu

**Keywords:** plague, cost-effectiveness, mass prophylaxis, doxycycline, insecticide

## Abstract

Plague (*Yersinia pestis*) remains endemic in certain parts of the world. We assessed the cost-effectiveness of plague control interventions recommended by the World Health Organization with particular consideration to intervention coverage and timing. We developed a dynamic model of the spread of plague between interacting populations of humans, rats, and fleas and performed a cost-effectiveness analysis calibrated to a 2017 Madagascar outbreak. We assessed three interventions alone and in combination: expanded access to antibiotic treatment with doxycycline, mass distribution of doxycycline prophylaxis, and mass distribution of malathion. We varied intervention timing and coverage levels. We calculated costs, quality-adjusted life years (QALYs), and incremental cost-effectiveness ratios from a healthcare perspective. The preferred intervention, using a cost-effectiveness threshold of $1350/QALY (GDP per capita in Madagascar), was expanded access to antibiotic treatment with doxycycline with 100% coverage starting immediately after the first reported case, gaining 543 QALYs at an incremental cost of $1023/QALY gained. Sensitivity analyses support expanded access to antibiotic treatment and leave open the possibility that mass distribution of doxycycline prophylaxis or mass distribution of malathion could be cost-effective. Our analysis highlights the potential for rapid expansion of access to doxycycline upon recognition of plague outbreaks to cost-effectively prevent future large-scale plague outbreaks and highlights the importance of intervention timing.

## 1. Introduction

The plague, caused by the bacterium *Yersinia pestis*, has caused some of humanity’s worst pandemics. In recent years, more focal epidemics have occurred in low- and middle-income countries, in some cases causing a substantial number of illnesses and deaths.

Plague can take many forms. Bubonic plague is a rodent disease transmitted to humans by infected fleas and causes swollen lymph nodes to appear as buboes on infected individuals [1]. Septicemic plague is a bacterial infection of the blood [2]. Pneumonic plague is a highly contagious respiratory disease with person-to-person droplet transmission. The 48-hour mortality rate of pneumonic plague is close to 100% if left untreated, so it is an important disease to treat and contain quickly [3]. It is possible for the bubonic plague to progress to more severe forms of plague.

In Madagascar, plague is endemic, with a number of bubonic cases reported every year. A 2017 outbreak caused an estimated 2417 cases and 209 deaths between 1 August and 4 December [4]. Over 75% of the cases were pneumonic, 15% were bubonic, one case was septicemic, and about 10% were unclassified [4]. Sixty-eight percent of cases occurred in the Analamanga region [4].

The World Health Organization (WHO) recommends two primary methods for plague mitigation [5]: vector control and antibiotic treatment. Malathion is recommended for rodent flea control [6]. Doxycycline, an inexpensive pill that can be rapidly and widely distributed, is a recommended antibiotic for confirmed and suspected cases of all forms of plague [7,8]. During plague outbreaks with many active pneumonic plague cases, antibiotic prophylaxis to individuals prior to exposure may be recommended [9,10].

Prior modeling studies of bubonic plague evaluated different model structures with interacting populations of humans, rats, and fleas [11,12]. One study developed a detailed model of bubonic plague but did not consider latent periods of exposure nor pneumonic and septicemic transmission [11]. A study of the 2017 Madagascar outbreak estimated the basic reproductive number and case fatality ratio using case counts [13]. Another study modeled the 2017 Madagascar outbreak using stochastic transmission models to estimate disease spread and epidemiological parameters [14]. More work is needed to understand the dynamics of the plague in Madagascar and elsewhere. Here we introduce a new model of bubonic, septicemic, and pneumonic plague that extends prior models [11,12]. Additionally, we go beyond disease dynamics and assess the effectiveness and cost-effectiveness of WHO-recommended interventions.

Little insight exists about the cost-effectiveness of different WHO-recommended interventions for plague or tradeoffs between intervention timing and effectiveness. Previous research has explored the cost-effectiveness of large-scale prophylaxis but has focused on other contagions (e.g., anthrax, *Helicobacter pylori*) [15,16]. Several analyses have discussed the importance of intervention timing [17,18] but have not evaluated cost-effectiveness nor assessed the tradeoff between high costs of rapid intervention versus low effectiveness of a late intervention.

We developed a dynamic model of plague that provides a useful and detailed framework for the evaluation of plague interventions. We used the model to assess the effectiveness and cost-effectiveness of three potentially complementary interventions—doxycycline treatment, doxycycline prophylaxis, and insecticide—that vary by coverage, implementation timing, and startup costs.

## 2. Methods

### 2.1. Model

**Figure 1:**
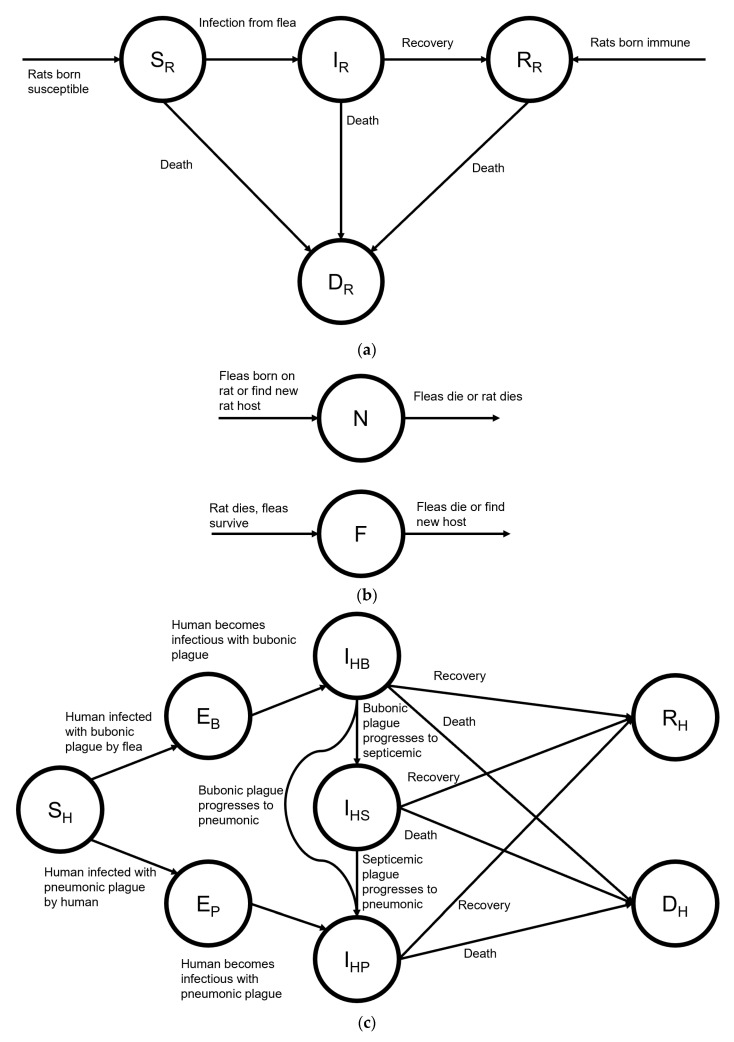
Schematic of a compartmental model of plague transmission among humans, rats, and fleas. The mathematics are described in Equations (S7)–(S15) in the Supplementary Materials. Figure S8 provides the transition rates. (**a**) SIR compartmental model for bubonic plague in rats. (**b**) Compartmental model for fleas carrying bubonic plague. (**c**) SEIR compartmental model for human plague transmission.

We developed a dynamic compartmental model of the spread of bubonic, septicemic, and pneumonic plague between interacting populations of humans, rats, and fleas using R (v4.0.2). Our modified SEIR model (Figure 1) extends prior models [11,12] and replicates the 2017 Madagascar outbreak. The model time horizon covers the duration of the outbreak in daily intervals over five months and captures the lifetime effects of disease and interventions on morbidity and mortality. The supplement provides full model details and a CHEERS checklist.

We instantiated the model for the Analamanga region of Madagascar. We relied primarily on data from previously published studies to estimate parameter values (Table 1). We found no data on progression rates from bubonic plague to septicemic or pneumonic plague or from septicemic to pneumonic plague and assumed they were at least 100 times less likely than natural transmission. We found no data on the infectious period of septicemic plague; we estimated this value as the mean of the infectious periods of bubonic and pneumonic plague [11,19].

We calibrated the model to WHO reports from October through December 2017, categorizing unknown cases as bubonic, septicemic, or pneumonic in proportion to known cases [4]. We assumed that the Analamanga region had the same proportion of bubonic, septicemic, and pneumonic cases as the country-wide total. To account for variability in disease transmission and recovery probability over the course of the outbreak, we divided the model timeline into four phases based on the WHO reports. We minimized the sum of root squared error to evaluate model accuracy during calibration. The Supplement provides full calibration details. Table 1 shows calibrated parameter values.

### 2.2. Interventions

We considered three interventions, alone and in combination, and considered different coverage levels and implementation timing.

Doxycycline treatment: Many public health organizations recommend doxycycline treatment and prophylaxis during pneumonic plague outbreaks [1,10,26,27,28]. Doxycycline treatment drastically reduces mortality and transmission rates [5]. Untreated bubonic, septicemic, and pneumonic plague have mortality probabilities of around 60%, 100%, and 100%, respectively [7,8,29]. The survival probability of plague-infected individuals treated with doxycycline in a randomized control trial in Tanzania was 97% [29]; the estimated survival probability for treated pneumonic and septicemic plague based on U.S. historical cases is 50–70% [30]. The case fatality ratio of the 2017 Madagascar outbreak was around 9% (much lower than the fatality rate in the absence of treatment), so we assumed there was an existing significant baseline level of antibiotic treatment during the outbreak as the WHO donated and delivered 1.2 million doses of antibiotics to Madagascar during the outbreak [4]. We set the maximum level for expanded antibiotic treatment equal to the number of deaths (142), assuming that deaths were due to lack of antibiotic treatment. We assumed that, once individuals receive treatment, their rate of transmission and progression to other forms of plague (if applicable) is reduced to zero [31]. We considered expanded antibiotic coverage levels of 10–100% in 10% increments of eligible individuals (i.e., those who did not previously receive treatment and would have otherwise died) after they become infectious. In the status quo, 0% of these eligible individuals were given treatment.

Doxycycline prophylaxis: We assumed that doxycycline prophylaxis is distributed to susceptible individuals at varying rates from 0 to 10,000 people/day in 1000 people/day increments. We assumed that the baseline survival probability on oral doxycycline was 97%, as with treatment [29,30]. Once individuals receive doxycycline prophylaxis, we assumed they take 100–200 mg doxycycline daily for the remainder of the outbreak or until they recover from any potential infection (symptoms present and then recede) [10]. Therefore, the number of doxycycline doses distributed in the modeled population can reach hundreds of millions. If an individual on doxycycline prophylaxis contracts plague, we assume the disease does not progress to pneumonic or septicemic plague.

Vector control: Vector control reduces the incidence of bubonic plague by increasing the flea mortality rate [5]. The WHO recommends both fenitrothion and malathion as an insecticide dust to control rodent fleas [6]. The 24-h flea mortality probability is 82% for fenitrothion and 75% for malathion [32,33]. According to the WHO, fenitrothion is considered moderately hazardous in concentrations of 20 g/kg, while malathion is considered only slightly hazardous in concentrations of 50 g/kg [6]. While both are recommended by the WHO, we chose to model vector control using malathion due to its lower likelihood of adverse effects. We transformed the mortality probability into a rate and added it to the baseline flea mortality rate. We modeled mass distribution of malathion to households, varying coverage levels from 10–100% in 10% increments, to capture different levels of compliance with household insecticide use. In every household covered by the intervention, fleas had increased mortality consistent with the mortality from malathion.

Intervention timing: We varied implementation timing for all interventions. We considered intervention start dates ranging from day 1, 2,…, 120. For clarity of presentation, we group them into rapid, early, and late intervention times. Rapid interventions start on days 1, 10, 20, 30, 40, 50, or 60 and continue throughout the remainder of the modeled time horizon. Early interventions start on days 65, 70, 80, or 90. Late interventions start on days 95, 100, 110, or 120. The different combinations of interventions and their timing and coverage lead to 19,951 different intervention possibilities. 

Cost-effectiveness Analysis: We took a healthcare system perspective and measured costs (2017 USD) and quality-adjusted life years (QALYs), both discounted at 3% [34]. We calculated incremental costs and QALYs gained for each intervention compared to the next best alternative.

Intervention costs (Table 2) include costs of personnel, administration, and equipment [35,36]. In the status quo, we considered the costs of training the 1800 community health workers (CHWs) and 300 doctors that were mentioned by the WHO situation reports, and of inpatient hospitalizations [4]. We assumed that additional CHWs are required for intervention scale-up. Each additional CHW receives the mean daily salary for community health workers in Madagascar, $4.58 [37]. The cost of inpatient hospital care is $9.17 per patient per day [38,39]. We assumed that hospitalization costs were incurred for 91% of infectious individuals, consistent with a 91% survival rate [4]. This is based on the assumption that hospital care results in antibiotic treatment and antibiotic treatment results in near-certain recovery.

Costs of expanded access to antibiotic treatment with doxycycline include costs of doxycycline and increased CHW staffing. The lowest price of generic doxycycline available in Burundi and Ethiopia is $0.014 per 100 mg pill [46]. The number of additional CHWs required to staff the program is a function of expanded access to treatment coverage. We used linear interpolation to determine the number of CHWs needed to improve survival rates by one percentage point. In a non-intervention setting, the assumed survival rate was the approximate historic untreated pneumonic plague case fatality ratio of 100%, and the number of CHWs is 0. In the status quo setting, the case fatality ratio was around 9%, the number of CHWs was 1800, and the number of doctors was 300. In other words, we create a linear function where the case fatality ratio is 100% with 0 CHWs and 9% with 2100 CHWs. All additional CHWs carry on throughout the remainder of the outbreak.

Previous programs for mass distribution of antibiotics in African countries informed our cost estimates for mass doxycycline distribution [47,48]. We estimated the administration cost per treated individual to be $1.61 [47]. We calculated drug-related costs on a per-dose basis.

For the vector control intervention, we assumed that the average household size is 4.7 individuals [45]. We used an estimated administration cost per household of $1.61 [47]. The cost of a container of 50% malathion insecticide is $4.63 [44].

Startup costs depended on intervention timing and coverage (details in Supplement) and included the costs of expanded access to additional CHWs, doxycycline doses, mass distribution administration per treated individual, and units of malathion. Startup costs decreased linearly with later intervention timing and increased linearly with intervention coverage [49].

After adjusting for the health-related quality of life (HRQoL) of Madagascar and discounting, we treated all deaths as incurring a loss of 23.64 QALYs (details in Supplement). We used an HRQoL of 0.38 for the duration of infection for individuals infected with plague based on utility values for respiratory H1N1 infection because of similarities in symptoms and severity [43].

The GDP per capita of Madagascar in 2017 USD is $450 [40]. The benchmarks we use to determine whether an intervention is likely to be considered cost-effective or very cost-effective are $1350/QALY (three times GDP per capita) and $450/QALY (GDP per capita), respectively [34].

### 2.3. Sensitivity Analysis

All costs and QALYs estimates are inexact, so we performed a wide range of sensitivity analyses. We performed one-way sensitivity analyses on key variables including cost of a daily doxycycline dose, cost of a unit of malathion, QALY loss per death, cost of mass prophylaxis administration, doxycycline efficacy, and adherence to doxycycline prophylaxis. Additionally, we developed a hypothetical outbreak model in which the disease spreads more slowly over a longer period (details in Supplement).

### 2.4. Patient and Public Involvement

It was not appropriate or possible to involve patients or the public in the design, or conduct, or reporting, or dissemination plans of our research.

## 3. Results

**Figure 2:**
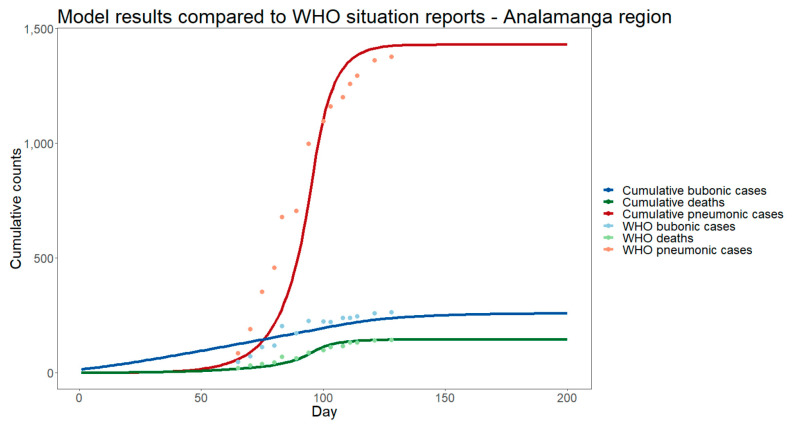
Calibrated model performance on cumulative pneumonic and bubonic plague cases and deaths in the Analamanga region of Madagascar.

Modeled cumulative case counts and deaths for bubonic and pneumonic cases from the calibrated model without additional intervention were within 10% of reported values [4] (Figure 2, Supplementary Materials Table S1). The root mean squared error of the model compared to the WHO reports was 104 for pneumonic cases, 38 for bubonic cases, and 10 for deaths. This was a mean absolute difference of 31%, 12%, and 14%, respectively, for pneumonic cases, bubonic cases, and deaths.

### 3.1. Base Case

**Figure 3:**
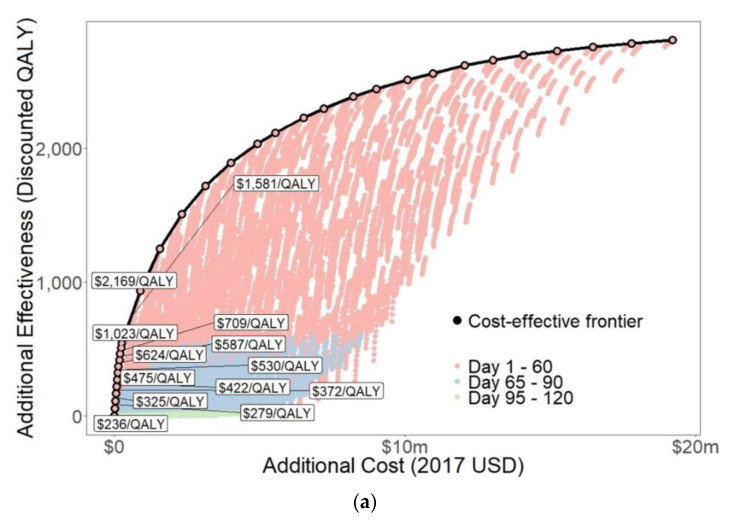

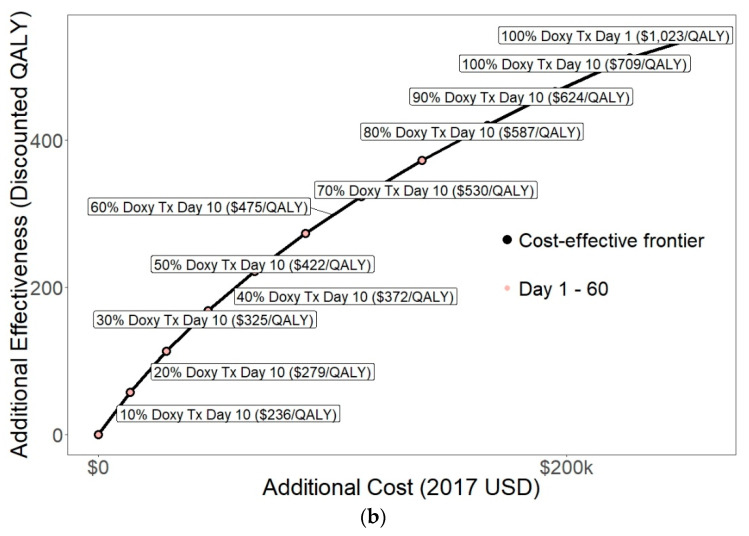
Cost-effectiveness frontier: (**a**). Full cost-effectiveness frontier; (**b**). Detailed view of cost-effectiveness frontier near the preferred decision.

Timing and type of intervention had a large impact on effectiveness (Figure 3, Table 3). Effectiveness consistently increased with coverage and decreased with longer intervention delays. Considering the intervention types singly, expanded access to antibiotic treatment with doxycycline gained the smallest range of QALYs, between 0.29–543 (Supplemental Figure S1). This is because the upper bound on expanded access to antibiotic coverage was limited by the roughly 140 deaths from the outbreak. This small cohort further limits downstream benefits of averted secondary infections. Mass distribution of doxycycline prophylaxis gained the largest range of QALYs, between 0.30–2331 (Figure S1), because it could prevent new infections and could cover a large portion of the population. Mass distribution of malathion gained between 0.47–1223 QALYs (Figure S1). 

Intervention costs varied by timing and coverage (Figure S2), generally decreasing as intervention implementation was delayed (Figure S2a) and increasing with coverage (Figure S2b–d). Costs of expanded access to antibiotic treatment with doxycycline ranged from $254 to $259,400 (Figure S2b). This was the cheapest intervention, as the number of recipients was relatively low. Costs of mass distribution of doxycycline prophylaxis ranged from $4497–$8.8 million, depending on implementation timing (Figure S1) and coverage (Figure S2c). Costs of mass distribution of malathion ranged from $155,400–$10.1 million (Figure S2d), depending mainly on intervention coverage.

Comparison of all 19,951 interventions resulted in a cost-effectiveness frontier with 33 interventions, including the status quo (Figure 3, Table 3). For a $450/QALY threshold, the preferred intervention is expanded access to antibiotic treatment with doxycycline at 50% coverage starting on day 10 ($422/QALY). For a $1350/QALY threshold, the preferred intervention is expanded access to antibiotic treatment with doxycycline at 100% coverage starting on day 1 ($1023/QALY) (Table 3).

**Figure 4:**
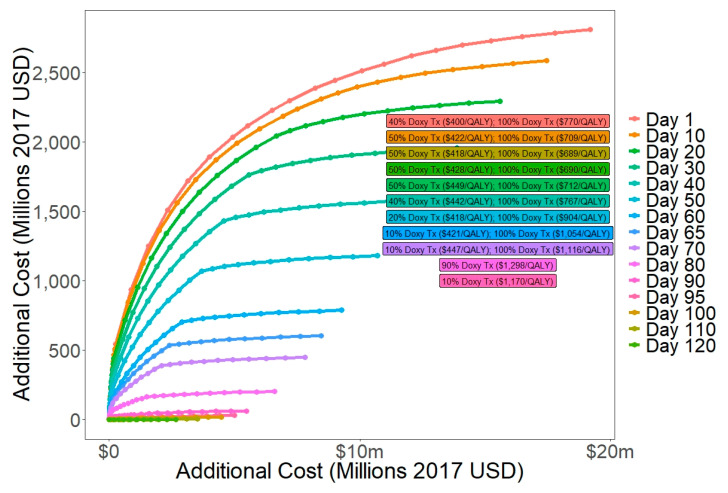
Cost-effectiveness frontiers when comparing single-day decisions.

We performed cost-effectiveness analyses on subsets of interventions with the same timing (Figure 4, Table S2). For interventions starting before day 80, the preferred intervention was expanded access to antibiotic treatment with doxycycline with 100% coverage which cost $689–1116/QALY gained. For interventions starting on days 80 and 90, the preferred intervention was expanded access to antibiotic treatment with doxycycline with 90% coverage ($1298/QALY) and 10% coverage ($1170/QALY), respectively. For implementation later than day 90, no interventions were cost-effective. This is likely because 61% of deaths and 75% of cases occurred before day 95, so late interventions have low potential effectiveness.

### 3.2. Sensitivity Analysis

When using a cost-effectiveness threshold of 1–51% of GDP, as recommended by some [50], then a strategy of expanded access to antibiotic treatment with doxycycline at 10% coverage implemented on day 10 is likely to be cost-effective.

In sensitivity analysis, varying adherence to and efficacy of doxycycline prophylaxis did not change the preferred intervention. 

Varying doxycycline cost per dose ($0.005–$0.02) only changed the preferred intervention at the lowest cost ($0.005) when the preferred intervention, costing $1002/QALY gained, was expanded access to antibiotic treatment at 100% coverage combined with the mass distribution of doxycycline prophylaxis at a rate of 1000 people/day. 

We varied the cost per unit of malathion between $0.50–$4.63. When the cost was $0.50, the preferred intervention, costing $1329/QALY gained, was expanded access to antibiotic treatment at 100% coverage combined with the mass distribution of malathion at 10% coverage. This suggests that if vector control is very cheap, mass distribution of malathion could be cost-effective.

We varied the QALY loss per death between 10–60 QALYs (Figure S3, Table S3). For fewer than 30 QALYs lost per death, the preferred intervention was expanded access to antibiotic treatment with coverage between 70–100%. For more than 30 QALYs lost per death, the preferred intervention also included the mass distribution of doxycycline prophylaxis at a rate of 1000–3000 people/day. This suggests the potential for mass distribution of doxycycline prophylaxis to be cost-effective.

We varied the administrative cost per individual of a mass doxycycline prophylaxis program between $0.25–$1.61 (Figure S4, Table S4). At $1.00 or less per person, the preferred intervention was the mass distribution of doxycycline prophylaxis at a rate of 1000–2000 people/day starting on day 1. This further supports the notion that mass distribution of doxycycline prophylaxis could be cost-effective under some conditions.

Our model can incorporate a wide range of policy constraints. For example, if the intervention cannot occur until 40 days after the first reported case, and coverage of each intervention is capped at 80%, then the preferred decision is expanded access to antibiotic treatment with 80% coverage starting on day 40, which costs $603/QALY (Figure S5).

Finally, we considered the case of a slower epidemic (Figure S6). We compared incremental costs and QALYs over the first 200 days of the simulated outbreak (Table S5, Figure S7). For a $450/QALY threshold, the preferred intervention is expanded access to antibiotic treatment with 100% coverage and mass distribution of doxycycline prophylaxis at a rate of 3000 people/day starting on day 110, which costs $431/QALY. For a $1350/QALY threshold, the preferred intervention is expanded access to antibiotic treatment at 100% coverage combined with the mass distribution of doxycycline prophylaxis at a rate of 8000 people/day starting on day 95, which costs $1252/QALY. This suggests that if an outbreak takes longer to develop, then the mass distribution of doxycycline prophylaxis could be cost-effective, and the preferred intervention will likely be delayed further.

## 4. Discussion

For an epidemic modeled on the 2017 Madagascar outbreak, we found that expanded access to antibiotic treatment with doxycycline is likely to be cost-effective at high coverage levels when implemented during the initial phase of the outbreak. Given the existing high level of antibiotic treatment during the outbreak, the low cost of expanding treatment to infected individuals not receiving antibiotics makes this intervention attractive. Mass distribution of malathion is unlikely to be cost-effective because of the relatively high cost per unit; additionally, this intervention primarily reduced bubonic plague, and the outbreak we modeled was largely pneumonic plague.

Mass distribution of doxycycline prophylaxis could be cost-effective if rapid intervention is comparable in cost to later intervention, or in a plague outbreak that develops more slowly than the 2017 outbreak. Mass doxycycline prophylaxis could be particularly useful in a country with weak healthcare infrastructure, such as Madagascar, as it is relatively simple to implement and does not require equipment or protocol for testing for plague because of the existing presence of treatment.

In Madagascar, annual federal government expenditures total just over $2 billion for a population where 70% of people live below the poverty line [26]. Therefore, the affordability of any healthcare intervention is an important consideration. The cost of the preferred intervention, increased treatment with doxycycline, is around $250,000 (0.0125% of the federal budget) suggesting that it is indeed affordable.

In countries with different cost-effectiveness thresholds but similar cost characteristics, the preferred intervention may differ. Plague is endemic in the Democratic Republic of Congo (DRC) [1,51]. GDP per capita on the DRC is approximately $460 [52]. Assuming an outbreak similar to the one we have modeled, expanded access to antibiotic treatment with doxycycline at 100% coverage implemented on day 1 is likely to be cost-effective in the DRC ($1023/QALY).

Our analysis has several limitations. Aside from the WHO situation report data, which was sparse and inconsistent, many parameter estimates in our model came from studies of plague that occurred in countries other than Madagascar and in different decades. This could affect the accuracy of the model projections for Madagascar and the model’s applicability to future outbreaks in the region. Additional research should focus on the disease and behavioral dynamics of Madagascar to better tailor the model to the local population and healthcare infrastructure. We calibrated to cumulative case counts of suspected, probable, and confirmed cases, which could underestimate the true disease burden due to underreporting or overreporting. With uncertainty in the true level of disease burden, our model could miss important QALY losses from unreported cases which would make the interventions we considered appear more cost-effective or may misattribute QALY losses from other diseases to those of plague, which would make the interventions appear less cost-effective. One study suggests that calibrating to cumulative cases may lead to errors in parameter estimates [53]. In addition, with only one reported case of septicemic plague during the outbreak we modeled, including septicemic plague in the model could overrepresent those cases in our analysis relative to their importance in the outbreak. Better surveillance, testing, and case reporting for plague are needed [54] and would help avoid these potential biases.

We made simplifying assumptions regarding certain clinical aspects of plague. For example, we assumed immediate reduction in transmission due to doxycycline treatment or prophylaxis and near-certain recovery for individuals who are hospitalized. In reality, these factors may depend on the delay from disease onset to treatment [55]. We assumed similar clinical presentation by region. We did not model subclinical plague infections which could be a driver for the persistence of plague during and between outbreaks. Subclinical infections are common in rat populations but rare in human populations [56,57]. We did not consider harms related to interventions such as side effects of doxycycline or potential health hazards of malathion and did not account for potential microbial resistance stemming from insecticide distribution or mass prophylaxis [58]. The potential harms from microbial or insecticide resistance could potentially outweigh the benefits of mass prophylaxis or mass insecticide distribution program in the long term.

Additionally, our model does not completely capture the realities of intervention implementation. We assumed a homogenous population that is well mixed and a homogeneous implementation of interventions. We assumed that interventions began at exact dates with exact coverages that remain at a constant level until the end of the outbreak. We have little idea how much it would cost to dramatically expand health coverage or deliver community-wide doxycycline over any period of time. During the 2017 plague outbreak, antibiotic prophylaxis was restricted to direct contacts of suspected, probable, and confirmed cases of plague [4]. The true feasibility of mass prophylaxis is unknown; however, the WHO donated more than 1 million doses of antibiotics during the 2017 outbreak [4]. Mass prophylaxis would be further constrained by limited access and potentially higher costs in rural areas. Finally, given that pneumonic plague dominated the 2017 outbreak and that *Yersinia pestis* can be used as a bioterror agent, it could be useful to explore additional interventions related to person-to-person droplet transmission.

Our model and recommendations are founded on retrospective data. In reality, intervention decisions will be made prospectively without knowing how big an outbreak will be. If Madagascar conducted mass distribution of doxycycline prophylaxis every time there was a bubonic plague case, it would be expensive and perhaps not cost-effective. Our sensitivity analysis with a slower epidemic showed that mass distribution of doxycycline prophylaxis could still be cost-effective if started at a later date. However, our analyses assume an outbreak that is primarily pneumonic plague. The most common endemic outbreaks will likely be largely or exclusively bubonic plague. Thus, pneumonic plague surveillance and detection are critical so that such outbreaks can be easily distinguished. For endemic bubonic plague, interventions related to rat control may need to be evaluated.

Our analysis aims to inform policymakers such as country-level decision-makers, international health organizations, and donor organizations about how to respond effectively and cost-effectively to plague outbreaks in Madagascar and elsewhere. This is important because the plague is endemic or epidemic in many countries, including the United States, and has been identified as a potential bioterror threat [3,8]. We found that expanded access to antibiotic treatment with doxycycline is cost-effective and mass distribution of doxycycline prophylaxis is potentially cost-effective in Madagascar, suggesting that these should be key foci for plague preparation and response in the future. Our work highlights the importance of intervention timing: early intervention is more effective than late intervention. However, if there are high startup costs for interventions early in the outbreak, then it is preferable to implement interventions around a month after the first reported case.

A recent article [59] notes that “Plans should be in place to reduce the risk of [plague] outbreaks and to manage and contain those that occur as swiftly as possible. These plans should be informed by recent experiences in Madagascar and Beijing, and assume that future outbreaks are not just likely but inevitable”. Our findings have the potential to improve future plague outbreak response strategies in Madagascar and elsewhere.

## Figures and Tables

**Table 1:** Plague Parameter Values and Sources.

*Parameter*	Description	Value	Source
*Initial Susceptible Population*	Sum of populations in districts with plague	13,731,412 people	[4]
$r_{R}$	Rat reproductive rate	5 rats/day	[11,20]
$K_{R}$	Rat carrying capacity	2500 rats	[11]
p	Probability of inherited resistance of rats	0.975	[11,21]
$\beta_{R}$	Transmission rate of bubonic plague to rats	4.7 contacts/(rat-day)	[11,22,23,24]
*α*	Flea searching efficiency	0.004	[11]
$m_{R}$	(Infectious period of bubonic plague in rats)^−1^	0.05 people/day	[11]
$d_{R}$	Death rate of rats	0.2 rats/day	[11,20,25]
$g_{R}$	Probability of recovery in rats	0.02	[11]
$r_{F}$	Flea reproductive rate	20 fleas/day	[11]
$K_{F}$	Flea carrying capacity per rat	6.57 fleas/rat	[11,21]
$d_{F}$	Death rate of fleas	10 fleas/day	[11,25]
α	Flea searching efficiency	0.004	[11]
$\varepsilon_{B}$	(Latency period of bubonic plague)^−1^	0.25 people/day	[11]
$\varepsilon_{P}$	(Latency period of pneumonic plague)^−1^	0.23 people/day	[19]
$\gamma_{\mathrm{BS}}$	Progression rate: bubonic to septicemic plague	0.001 people/day	Assumed
$\gamma_{\mathrm{BP}}$	Progression rate: bubonic to pneumonic plague	0.001 people/day	Assumed
$\gamma_{\mathrm{SP}}$	Progression rate: septicemic to pneumonic plague	0.001 people/day	Assumed
$m_{\mathrm{HB}}$	(Infectious period of bubonic plague in humans)^−1^	0.04 people/day	[11]
$m_{\mathrm{HS}}$	(Infectious period of septicemic plague)^−1^	0.07 people/day	Assumed
$m_{\mathrm{HP}}$	(Infectious period of pneumonic plague)^−1^	0.4 people/day	[19]
$g_{\mathrm{HS}}$	Probability of recovery from septicemic plague	0.8	[2,4,8]
$\beta_{\mathrm{HB}}$	Transmission rate of bubonic plague to humans	Phase 1–3: 0.2 contacts/(person-day)	Calibrated
		Phase 4: 0 contacts/(person-day)	
$\beta_{\mathrm{HP}}$	Transmission rate of pneumonic plague between humans at each phase (step)	Phase 1: 0.64 contacts/(person-day)	Calibrated
		Phase 2: 0.68 contacts/(person-day)	
		Phase 3: 0.10 contacts/(person-day)	
		Phase 4: 0 contacts/(person-day)	
$g_{\mathrm{HB}}$	Probability of recovering from bubonic plague at each phase (step)	Phase 1: 0.90	Calibrated
		Phase 2: 0.91	
		Phase 3: 0.94	
		Phase 4: 0.99	
$g_{\mathrm{HP}}$	Probability of recovering from pneumonic plague at each phase (step)	Phase 1: 0.81	Calibrated
		Phase 2: 0.9	
		Phase 3: 0.93	
		Phase 4: 0.99	

**Table 2:** Cost and Utility Parameter Values and Sources.

Parameter Name/Description	Parameter Value	Source
**Median age**	19.9	[40]
**Median life expectancy at birth**	66.6	[40]
**Quality-adjusted life expectancy**	55.0	[41]
**Mean life expectancy at age 20**	50.6	[42]
**HRQoL during infection**	0.38	[43]
**Cost of inpatient hospitalization per patient per day**	$9.17	[39]
**Mean daily salary for community health workers**	$4.58	[37]
**Container of 50% malathion insecticide**	$4.63	[44]
**Average household size**	4.7	[45]
**Price of generic doxycycline per daily dose**	$0.014	[46]
**Overhead cost per individual receiving treatment**	$0.32	[47]
**Overhead costs per individual receiving prophylaxis**		
**Vehicles**	$0.05	[47]
**Communication and IT equipment**	$0.01	[47]
**Mass distribution equipment**	$0.02	[47]
**Travel and transportation**	$0.39	[47]
**Vehicle fuel and maintenance**	$0.03	[47]
**Accommodation and sustenance**	$0.13	[47]
**Mass distribution consumables and other charges**	$0.08	[47]
**Communication**	$0.01	[47]
**Personnel**	$0.57	[47]
**Total**	$1.61	
**Annual discount rate**	3%	[34]

HRQoL = health-related quality of life; IT = information technology.

**Table 3:** Costs and Effectiveness of Interventions on the Cost-Effectiveness Frontier.

Intervention Timing	Doxycycline Treatment Expanded Coverage	Doxycycline Prophylaxis Distribution Rate, People/Day (Final Coverage as % of Total Population)	Malathion Distribution Coverage	Total Cost	Total QALYs	Incremental Cost	Incremental QALYs	ICER (Cost/QALY Gained)
N/A	0%	0 (0%)	0%	$0	0	N/A	N/A	N/A
Day 10	10%	0 (0%)	0%	$13,620	57.7	$13,620	57.7	$236
Day 10	20%	0 (0%)	0%	$29,290	113.9	$15,670	56.2	$279
Day 10	30%	0 (0%)	0%	$47,000	168.4	$17,710	54.5	$325
Day 10	40%	0 (0%)	0%	$66,750	221.5	$19,750	53.1	$372
Day 10	50%	0 (0%)	0%	$88,550	273.1	$21,800	51.6	$422
Day 10	60%	0 (0%)	0%	$112,400	323.3	$23,840	50.2	$475
Day 10	70%	0 (0%)	0%	$138,300	372.1	$25,880	48.8	$530
Day 10	80%	0 (0%)	0%	$166,200	419.7	$27,920	47.6	$587
Day 10	90%	0 (0%)	0%	$195,000	465.9	$28,850	46.2	$624
Day 10	100%	0 (0%)	0%	$227,000	511.0	$31,950	45.1	$709
Day 1	100%	0 (0%)	0%	$259,400	542.7	$32,380	31.7	$1023
Day 1	100%	1000 (%)	0%	$879,600	934.9	$620,200	392.2	$1581
Day 1	100%	2000 (%)	0%	$1,567,000	1252	$687,600	317.1	$2169
Day 1	100%	3000 (%)	0%	$2,318,000	1509	$751,100	257.5	$2917
Day 1	100%	4000 (%)	0%	$3,129,000	1720	$811,000	210.3	$3857
Day 1	100%	5000 (%)	0%	$3,997,000	1892	$867,400	172.6	$5027
Day 1	100%	6000 (%)	0%	$4,917,000	2035	$920,600	142.4	$6465
Day 1	100%	6000 (%)	10%	$5,529,000	2115	$612,100	80.7	$7584
Day 1	100%	7000 (%)	10%	$6,500,000	2228	$970,800	112.1	$8658
Day 1	100%	7000 (%)	20%	$7,201,000	2295	$701,200	68.0	$10,310
Day 1	100%	8000 (%)	20%	$8,220,000	2384	$1,018,00	88.7	$11,470
Day 1	100%	8000 (%)	30%	$9,010,000	2442	$790,300	57.3	$13,790
Day 1	100%	9000 (%)	30%	$10,070,000	2512	$1,063,000	70.6	$15,060
Day 1	100%	9000 (%)	40%	$10,950,000	2560	$879,200	48.3	$18,200
Day 1	100%	10,000 (%)	40%	$12,060,000	2617	$1,105,000	56.4	$19,570
Day 1	100%	10,000 (%)	50%	$13,020,000	2658	$968,200	40.8	$23,760
Day 1	100%	10,000 (%)	60%	$14,080,000	2694	$1,057,000	36.5	$28,930
Day 1	100%	10,000 (%)	70%	$15,230,000	2727	$1,146,000	32.7	$35,000
Day 1	100%	10,000 (%)	80%	$16,460,000	2756	$1,234,000	29.4	$42,050
Day 1	100%	10,000 (%)	90%	$17,780,000	2783	$1,323,000	26.4	$50,200
Day 1	100%	10,000 (%)	100%	$19,200,000	2806	$1,412,000	23.7	$59,540

ICER = incremental cost-effectiveness ratio, QALY = quality-adjusted life year.

## Data Availability

Data are available at https://www.afro.who.int/health-topics/plague/plague-outbreak-situation-reports (accessed on 21 February 2018).

## Supplementary Materials

The following are available online at https://www.mdpi.com/article/10.3390/tropicalmed6020101/s1, Figure S1: QALYs gained for different interventions as a function of implementation timing. The interventions plotted include only those which have one intervention type, Figure S2: Intervention costs as a function of implementation timing and coverage, Figure S3: Sensitivity analysis on QALYs lost per death for cost-effective and very cost-effective interventions, Figure S4: Sensitivity analysis on the administrative cost per person of mass prophylaxis distribution program for cost-effective and very cost-effective interventions, Figure S5: Cost-effectiveness frontier when decision is restricted to interventions day 40 or later where coverage of additional antibiotic treatment is 80% or less and coverage of mass distribution of doxycycline prophylaxis or of malathion is 80% or less, Figure S6. In sensitivity analysis, we developed a model of an outbreak that spreads more slowly than the actual 2017 outbreak, Figure S7. Cost-effectiveness frontier for slower epidemic, Figure S8: SEIR compartmental model for human plague transmission showing transition parameters, Table S1: Calibrated Model Projections Compared to WHO Situation Reports, Percent Difference from Adjusted WHO Values, Table S2: Costs and Effectiveness of Cost-Effective Interventions for Different Intervention Implementation Days, Table S3: Costs and Effectiveness of Interventions on the Cost-Effectiveness Frontier for Different QALY Loss per Death, Table S4: Costs and Effectiveness of Interventions on the Cost-Effectiveness Frontier for Different Administrative Cost per Person of Mass Distribution, Table S5: Costs and Effectiveness of Interventions on the Cost-Effectiveness Frontier for a Slower Epidemic, detailed description of dynamic transmission model, description of calculation of health-related quality of life, description of sensitivity analyses with slower epidemic model, CHEERS Checklist.

## References

[1] Centers for Disease Control and Prevention Recommended Antibiotic Treatment for Plague. https://www.cdc.gov/plague/resources/Recommended-antibiotics-for-plague_revision-Aug-2015_Final-(00000002).pdf.

[2] Hull H.F., Montes J.M., Mann J.M. (1987). Septicemic plague in New Mexico. J. Infect. Dis..

[3] Riedel S. (2005). Plague: From natural disease to bioterrorism. Bayl. Univ. Med. Cent. Proc..

[4] World Health Organization Plague Outbreak Madagascar, External Situation Report 14. https://www.afro.who.int/health-topics/plague/plague-outbreak-situation-reports.

[5] World Health Organization (2006). Interregional meeting on prevention and control of plague. Epidemic and Pandemic Alert and Response.

[6] World Health Organization (2006). Pesticides and Their Application: For the Control of Vectors and Pests of Public Health Importance.

[7] Brouillard J.E., Terriff C.M., Tofen A., Garrison M.W. (2006). Antibiotic selection and resistance issues with fluoroquinolones and doxycycline against bioterrorism agents. Pharmacotherapy.

[8] Inglesby T.V., Dennis D.T., Henderson D.A., Bartlett J.G., Ascher M.S., Eitzen E., Fine A.D., Friedlander A.M., Hauer J., Koerner J.F. (2000). Plague as a biological weapon. JAMA.

[9] Centers for Disease Control and Prevention (1996). Prevention of plague: Recommendations of the Advisory Committee on Immunization Practices (ACIP). MMWR.

[10] World Health Organization Plague Manual: Epidemiology, Distribution, Surveillance and Control. https://www.who.int/csr/resources/publications/plague/WHO_CDS_CSR_EDC_99_2_EN/en/.

[11] Keeling M.J., Gilligan C.A. (2000). Bubonic plague: A metapopulation model of a zoonosis. Proc. Biol. Sci..

[12] Keeling M.J., Gilligan C.A. (2000). Metapopulation dynamics of bubonic plague. Nature.

[13] Tsuzuki S., Lee H., Miura F., Chan Y.H., Jung S.M., Akhmetzhanov A.R., Nishiura H. (2017). Dynamics of the pneumonic plague epidemic in Madagascar, August to October 2017. Eurosurveillance.

[14] Nguyen V.K., Parra-Rojas C., Hernandez-Vargas E.A. (2018). The 2017 plague outbreak in Madagascar: Data descriptions and epidemic modelling. Epidemics.

[15] Fowler R.A., Sanders G.D., Bravata D.M., Nouri B., Gastwirth J.M., Peterson D., Broker A.G., Garber A.M., Owens D.K. (2005). Cost-effectiveness of defending against bioterrorism: A comparison of vaccination and antibiotic prophylaxis against anthrax. Ann. Intern. Med..

[16] Rupnow M.F., Chang A.H., Shachter R.D., Owens D.K., Parsonnet J. (2009). Cost-effectiveness of a potential prophylactic *Helicobacter pylori* vaccine in the United States. J. Infect. Dis..

[17] Cuong H.Q., Vu N.T., Cazelles B., Boni M.F., Thai K.T., Rabaa M.A., Quang L.C., Simmons C.P., Huu T.N., Anders K.L. (2013). Spatiotemporal dynamics of dengue epidemics, southern Vietnam. Emerg. Infect. Dis..

[18] Teklehaimanot H.D., Schwartz J., Teklehaimanot A., Lipsitch M. (2004). Weather-based prediction of *Plasmodium falciparum* malaria in epidemic-prone regions of Ethiopia II. Weather-based prediction systems perform comparably to early detection systems in identifying times for interventions. Malar. J..

[19] Gani R., Leach S. (2004). Epidemiological determinants for modeling pneumonic plague outbreaks. Emerg. Infect. Dis..

[20] Buckle A.P., Smith R.H. (1994). Rodent Pests and Their Control.

[21] Hirst L.F. (1938). Plague. British Encyclopedia of Medical Practice, Volume 9.

[22] Hinnebusch B.J., Gage K.L., Schwan T.G. (1998). Estimation of vector infectivity rates for plague by means of a standard curve-based competitive polymerase chain reaction method to quantify *Yersinia pestis* in fleas. Am. J. Trop. Med. Hyg..

[23] Macchiavello A. (1954). Reservoirs and vectors of plague. J. Trop. Med. Hyg..

[24] Wheeler C.M., Douglas J.R. (1945). Sylvatic plague studies: V. The determination of vector efficiency. J. Infect. Dis..

[25] Bacot A.W. (1915). Observations on the length of time that fleas (*Ceratophyllus fasciatus*) carrying *Bacillus pestis* in the alimentary canals are able to survive in the absence of a host and retain the power to re-infect with the plague. J. Hyg..

[26] Central Intelligence Agency The World Factbook: Madagascar. https://www.cia.gov/library/publications/the-world-factbook/geos/ma.html.

[27] New Mexico Department of Health Mass Post Exposure Prophylaxis Protocol. https://nmhealth.org/publication/view/policy/2964/.

[28] Oklahoma State Department of Health Plague Prophylaxis (Bioterrorism). https://www.ok.gov/health2/documents/Plague%20Prophylaxis.pdf.

[29] Narayanan N., Lacy C.R., Cruz J.E., Nahass M., Karp J., Barone J.A., Hermes-DeSantis E.R. (2018). Disaster preparedness: Biological threats and treatment options. Pharmacotherapy.

[30] Mwengee W., Butler T., Mgema S., Mhina G., Almasi Y., Bradley C., Formanik J.B., Rochester C.G. (2006). Treatment of plague with gentamicin or doxycycline in a randomized clinical trial in Tanzania. Clin. Infect. Dis..

[31] Hinckley A.F., Biggerstaff B.J., Griffith K.S., Mead P.S. (2012). Transmission dynamics of primary pneumonic plague in the USA. Epidemiol. Infect..

[32] Miarinjara A., Vergain J., Kavaruganda J.M., Rajerison M., Boyer S. (2017). Plague risk in vulnerable community: Assessment of *Xenopsylla cheopis* susceptibility to insecticides in Malagasy prisons. Infect. Dis. Poverty.

[33] Rajonhson D.M., Miarinjara A., Rahelinirina S., Rajerison M., Boyer S. (2017). Effectiveness of fipronil as a systemic control agent against *Xenopsylla cheopis* (*Siphonaptera: Pulicidae*) in Madagascar. J. Med. Entomol..

[34] Gold M.R., Siegel J.E., Russell L.B., Weinstein M.C. (1996). Cost-Effectiveness in Health and Medicine.

[35] Goldhaber-Fiebert J.D., Denny L.A., De Souza M., Kuhn L., Goldie S.J. (2009). Program spending to increase adherence: South African cervical cancer screening. PLoS ONE.

[36] Goldhaber-Fiebert J.D., Denny L.E., De Souza M., Wright T.C., Kuhn L., Goldie S.J. (2005). The costs of reducing loss to follow-up in South African cervical cancer screening. Cost Eff. Resour. Alloc..

[37] Votresalaire.org Controle des Salaires [Wage Control]. https://votresalaire.org/madagascar/salaire/controle-des-salaires#/.

[38] Lee B.Y., Brown S.T., Haidari L.A., Clark S., Abimbola T., Pallas S.E., Wallace A.S., Mitgang E.A., Leonard J., Bartsch S.M. (2019). Economic value of vaccinating geographically hard-to-reach populations with measles vaccine: A modeling application in Kenya. Vaccine.

[39] World Health Organization WHO-CHOICE Estimates of Cost for Inpatient and Outpatient Health Service Delivery. https://www.who.int/choice/cost-effectiveness/inputs/health_service/en/.

[40] The World Bank Madagascar. https://data.worldbank.org/country/madagascar.

[41] World Health Organization Madagascar: WHO Statistical Profile. https://www.who.int/gho/countries/mdg/data/en/.

[42] World Health Organization Life Tables by Country: Madagascar. http://apps.who.int/gho/data/?theme=main&vid=60970.

[43] Hollmann M., Garin O., Galante M., Ferrer M., Dominguez A., Alonso J. (2013). Impact of influenza on health-related quality of life among confirmed (H1N1) 2009 patients. PLoS ONE.

[44] World Health Organization Sources and Prices of Selected Products for the Prevention, Diagnosis and Treatment of Malaria. https://apps.who.int/iris/handle/10665/43080.

[45] United Nations Household Size and Composition around the World 2017. https://www.un.org/en/development/desa/population/publications/pdf/ageing/household_size_and_composition_around_the_world_2017_data_booklet.pdf.

[46] Health Action International Price and Availability for Doxycycline 100 mg cap/Table. http://www.haiweb.org/MedPriceDatabase/price_availability_medicine_main.php?MP_ID=0&MED_ID=1364.

[47] Kolaczinski J.H., Robinson E., Finn T.P. (2011). The cost of antibiotic mass drug administration for trachoma control in a remote area of South Sudan. PLoS Negl. Trop. Dis..

[48] Goldman A.S., Guisinger V.H., Aikins M., Amarillo M.L., Belizario V.Y., Garshong B., Gyapong J., Kabali C., Kamal H.A., Kanjilal S. (2007). National mass drug administration costs for lymphatic filariasis elimination. PLoS Negl. Trop. Dis..

[49] Johns B., Baltussen R., Hutubessy R. (2003). Programme costs in the economic evaluation of health interventions. Cost Eff. Resour. Alloc..

[50] Woods B., Revill P., Sculpher M., Claxton K. (2016). Country-level cost-effectiveness thresholds: Initial estimates and the need for further research. Value Health.

[51] World Health Organization Plague. https://www.who.int/news-room/fact-sheets/detail/plague.

[52] The World Bank Congo, Democratic Republic. https://data.worldbank.org/country/congo-dem-rep.

[53] King A.A., Domenech de Celles M., Magpantay F.M., Rohani P. (2015). Avoidable errors in the modelling of outbreaks of emerging pathogens, with special reference to Ebola. Proc. Biol. Sci..

[54] Mead P.S. (2018). Plague in Madagascar—A tragic opportunity for improving public health. N. Engl. J. Med..

[55] Andrianaivoarimanana V., Piola P., Wagner D.M., Rakotomanana F., Maheriniaina V., Andrianalimanana S., Chanteau S., Rahalison L., Ratsitorahina M., Rajerison M. (2019). Trends of human plague, Madagascar, 1998–2016. Emerg. Infect. Dis..

[56] European Centre for Disease Prevention and Control Factsheet about Plague. https://ecdc.europa.eu/en/plague/facts.

[57] New Mexico Department of Health Epidemiology and Response Division Plague. https://nmhealth.org/publication/view/help/1009/.

[58] Cabanel N., Bouchier C., Rajerison M., Carniel E. (2018). Plasmid-mediated doxycycline resistance in a Yersinia pestis strain isolated from a rat. Int. J. Antimicrob. Agents.

[59] Ranaivozanany D., Renaud B., Lucey D. (2020). Containing pneumonic plague. BMJ.

