# Supplementary material for: Modeling the Cost-Effectiveness of Interventions to Prevent Plague in Madagascar
Source: Trop Med Infect Dis. 2021 Jun 11;6(2):101. doi: 10.3390/tropicalmed6020101 (PMC8293333; doi:10.3390/tropicalmed6020101)
Supplement: Supplementary file 1 [file tropicalmed-06-00101-s001.zip › tropicalmed-1252796-supplementary.pdf]

Supplement: Cost-Effectiveness of Interventions to Prevent Plague in Madagascar

Giovanni S.P. Malloy, Margaret L. Brandeau and Jeremy D. Goldhaber-Fiebert

This supplement presents full details of our model and cost functions and health-related quality of life calculations, details of a hypothetical plague outbreak that develops more slowly than the 2017 Madagascar outbreak, and supplemental results.

Model Details

Our modified SEIR model extends prior models[1 2] and replicates the 2017 plague outbreak in Madagascar (Figure 1). The model time horizon covers the duration of the outbreak in daily intervals and captures the lifetime effects of the disease and of intervention on morbidity and mortality. The model includes three interacting populations: rats, fleas, and humans. Table 1 defines the model parameters.

Rats are either susceptible (S_R), infected (I_R), recovered (R_R), or dead (D_R) (Figure 1a). Susceptible rats are born into the population and are infected by fleas carrying bubonic plague. Some rats are born immune to bubonic plague. We assume that infected rats do not reproduce. All rats have a daily mortality risk. Equations (1) – (4) model the rat population.

$$\frac{dS_R}{dt} = r_R S_R\left(1 - \frac{T_R}{K_R}\right) + r_R R_R(1-p) - \beta_R\left(\frac{S_R}{T_R}\right)F\left(1 - e^{-\alpha T_R}\right) - d_R S_R \quad (1)$$

$$\frac{dI_R}{dt} = \beta_R\left(\frac{S_R}{T_R}\right)F\left(1 - e^{-\alpha T_R}\right) - (m_R + d_R)I_R \quad (2)$$

$$\frac{dR_R}{dt} = r_R R_R\left(p - \frac{T_R}{K_R}\right) + m_R g_R I_R - d_R R_R \quad (3)$$

$$T_R = S_R + I_R + R_R \quad (4)$$

$where\ S_R \geq 0, I_R \geq 0, R_R \geq 0$

Fleas are either on a rat or searching for a new rat or human host (Figure 1b). N is the average number of fleas on each rat. Each flea stays on that rat until either the flea or the rat dies. When rats die and the fleas on those rats survive, we scale the average fleas per rat by dead rats per day to determine the number of fleas that move to a second compartment, F: the total number of fleas looking for a host. Equations (5) and (6) model the flea population.

$$\frac{dN}{dt} = r_F N\left(1 - \frac{N}{K_F}\right) - \left(\frac{d_F}{T_R}\right)F\left(1 - e^{-\alpha T_R}\right) \quad (5)$$

$$\frac{dF}{dt} = \left(d_R + m_R(1 - g_R)\right)I_R N - d_F F \quad (6)$$

$where\ N\ \geq 0, F\ \geq 0$

Humans are either susceptible (S_R), exposed to bubonic or pneumonic plague ($E_B,\ E_P$, respectively), infected with bubonic, septicemic, or pneumonic plague ($I_{HB},\ I_{HS},\ I_{HP}$, respectively), recovered (R_H), or dead (D_H) (Figure 1c). The entire population of 3,348,794 individuals starts as susceptible. If a human is bitten by an infected flea, there is a latent period when the individual is infected but not symptomatic or infectious. The latent period for pneumonic plague is shorter. Because septicemic plague was rare during the outbreak, the model does not include septicemic plague transmission.[3] Once an individual becomes infectious with bubonic plague, the person may progress to septicemic or pneumonic plague, recover from the disease, or die. An individual who progresses to septicemic plague can progress further to pneumonic plague, recover, or die. Pneumonic plague infections end in either death or recovery. Because the time horizon of the model is less than a year, the human model does not include births into the population nor deaths from causes other than plague. Equations (7) – (15) govern the human population.

$$\frac{dS_H}{dt} = -\beta_{HB}\frac{S_H}{T_H}F(e^{-\alpha T_R}) - \beta_{HP}\left(\frac{S_H}{T_H}\right)I_{HP} \tag{7}$$

$$\frac{dE_B}{dt} = \beta_{HB}\frac{S_H}{T_H}F(e^{-\alpha T_R}) - \varepsilon_B E_B \tag{8}$$

$$\frac{dE_P}{dt} = \beta_{HP}\frac{S_H}{T_H}I_{HP} - \varepsilon_P E_P \tag{9}$$

$$\frac{dI_{HB}}{dt} = \varepsilon_B E_B - \gamma_{BS}I_{HB} - \gamma_{BP}I_{HB} - m_{HB}I_{HB} \tag{10}$$

$$\frac{dI_{HS}}{dt} = \gamma_{BS}I_{HB} - \gamma_{SP}I_{HS} - m_{HS}I_{HS} \tag{11}$$

$$\frac{dI_{HP}}{dt} = \varepsilon_P E_P + \gamma_{BP}I_{HB} + \gamma_{SP}\mathrm{I}_{HS} - m_{HP}I_{HP} \tag{12}$$

$$\frac{dR_H}{dt} = m_{HB}g_{HB}I_{HB} + m_{HS}g_{HS}I_{HS} + m_{HP}g_{HP}I_{HP} \tag{13}$$

$$\frac{dD_H}{dt} = m_{HB}(1-g_{HB})I_{HB} + m_{HS}(1-g_{HS})I_{HS} + m_{HP}(1-g_{HP})I_{HP} \tag{14}$$

$$T_H = S_H + E_{HB} + E_{HP} + I_{HB} + I_{HS} + I_{HP} + R_H + D_H \tag{15}$$

$$where\ S_H \geq 0, E_B \geq 0, E_P \geq 0, I_{HB} \geq 0, I_{HS} \geq 0, I_{HP} \geq 0, R_H \geq 0, D_H \geq 0$$

Model Instantiation

We relied primarily on data from previously published studies to estimate parameter values (Table 1). The rat reproductive rate (r_R), probability of inherited resistance (p), transmission rate of bubonic plague to rats (β_R), rat death rate (d_R), flea carrying capacity per rat (K_F), flea death rate (d_R), and flea movement rate (μ_F) were all obtained from either laboratory experiments or field observations.[1 4-8] We found no data on the progression rate of bubonic plague to septicemic plague (γ_{BS}), progression rate of bubonic plague to pneumonic plague (γ_{BP}), progression rate of septicemic plague to pneumonic plague (γ_{SP}), nor the infectious period of septicemic plague (m_{SH}). We estimated the infectious period of septicemic plague in humans by computing the mean of the infectious period of bubonic plague in humans and the infectious period of pneumonic plague.[1 9]

We used R to build and calibrate the model and to complete the analysis.

Model Calibration

We calibrated the model to WHO Situation Reports[3] with unknown cases categorized as bubonic, septicemic, or pneumonic in proportion to known cases. We assumed that the Analamanga region has the same proportion of bubonic, septicemic, and pneumonic cases as the country-wide total.

To account for variability in parameter values over the course of the outbreak, we divided the model timeline into four phases based on the WHO situation reports. The baseline response to the outbreak improved over time. These phases allowed us to calibrate separate transmission rates for different periods: in particular, the transmission rate of pneumonic plague, the probability of recovery from bubonic plague, and the probability of recovery from pneumonic plague.

1. August 1, 2017 – October 4, 2017 (Day 1 – 64): No WHO situation reports released. We assume that the disease spread without intervention during this period.

2. October 5, 2017 – October 31, 2017 (Day 65 – 94): WHO situation reports start to be released. The response focused on developing and beginning to implement a response plan. At the end of the period only about 25% of the planned response was funded.

3. November 1, 2017 – December 4, 2017 (Day 95 – 127): WHO response improves. The situation reports describe a decline in incidence and a focus on maintaining response rather than ramping up response.

4. December 5 – End of Outbreak (Day 128 –): No additional transmission

The result is a step function for each of the parameters to be calibrated. The changes in parameters over time reflect the improvement in treatment and mitigation of the disease.

For each proposed transmission rate and recovery probability parameter, we evaluated the accuracy of the model using the sum of root squared errors:

$$min \sum_i \sqrt{(WHO_i - Model_i)^2} \qquad (16)$$

We then chose the parameter value which minimized this value. For phase 1, there were no reports to compare against, so we calibrated to the first situation report. For phase 2, there were six situation reports, and for phase 3, there were seven. The final cumulative case and deaths counts were the calibration targets of phase 4.

We first estimated the transmission rates (β_{HB} and β_{HP}) for each phase.[3] We determined the total cumulative bubonic case counts from the model at day i from the number of individuals who recovered and died from bubonic plague at day $j = i + 1/m_{HB} + 3$. Day j accounts for the mean length of infection and a reporting delay.[10] We assumed that the transmission rate of bubonic plague is constant over the course of the first

three phases of the outbreak. This is reasonable because the biting rates of fleas, rather than human-to-human interaction, determine bubonic plague infection rates in rats and humans. We calibrated the transmission rate β_{HB} before β_{HP} because cases of bubonic cases can progress to cases of pneumonic plague and therefore affect the cumulative number of pneumonic cases. We used a similar calibration approach to determine the transmission rate of pneumonic plague. We accounted for reporting delays in the same manner; however, the transmission rate of pneumonic plague varies for each of the first three phases of the model.

Lastly, we calibrated the probabilities of recovery from bubonic and pneumonic plague (g_{HB} and g_{HP}). We compared the cumulative number of deaths reported by the WHO at day i to that of the model at day $k = i + 3$, which accounts for delays in reporting. Since the death counts reported by the WHO did not distinguish deaths by form of plague, we compared the WHO death counts to total deaths from all forms of plague from the model and assumed $g_{HB} > g_{HP}$. The calibrated parameter values are shown in Table 1.

Interventions

Many public health organizations recommend doxycycline distribution during pneumonic plague outbreaks.[11-13] Doxycycline treatment drastically reduces mortality and transmission rates.[14] To model this impact, we assumed that every person who died from plague did not receive antibiotics. Historically, untreated pneumonic plague has a case fatality ratio close to 100%.[15 16] Increased doxycycline treatment increases the survival probability of those who otherwise would have died. The survival probability of infected individuals treated with doxycycline is 97%.[17 18] To determine how many individuals would have otherwise died, we start at day j and run the model forward three days corresponding to the infectious period of the disease. We count the number of deaths at day $j + 3$ and determine which portion of the infected population on day j is eligible for doxycycline based on the level of coverage. Finally, we run the model again starting at day j to increment one day and repeat the process for every day in the simulation. Importantly, once individuals are put on treatment, their rate of transmission and disease progression is reduced to zero; this assumption reflects the lack of secondary infections from pneumonic plague cases in the United States in the antibiotic era.[19] We considered antibiotic coverage levels of 10-100% of eligible individuals (i.e., those who did not previously receive treatment and would have otherwise died) in 10% increments.

We implement doxycycline distribution in the model by replacing equations (10), (11), and (12) with equations (10a), (11a), (12a), (10b), (11b), and (12b), where τ_i is the proportion of the population infected with disease form *i* eligible for doxycycline treatment, and equations (13) and (14) are replaced by equations (13a) and (14a).

$$\frac{dI_{HB}}{dt} = (1 - \tau_B)\varepsilon_B E_B - \gamma_{BS} I_{HB} - \gamma_{BP} I_{HB} - m_{HB} I_{HB} \tag{10a}$$

$$\frac{dI_{HS}}{dt} = (1 - \tau_S)\gamma_{BS} I_{HB} - \gamma_{SP} I_{HS} - m_{HS} I_{HS} \tag{11a}$$

$$\frac{dI_{HP}}{dt} = (1 - \tau_P)(\varepsilon_P E_P + \gamma_{BP} I_{HB} + \gamma_{SP} I_{HS}) - m_{HP} I_{HP} \tag{12a}$$

$$\frac{dI_{TXHB}}{dt} = \tau_B \varepsilon_B E_B - m_{TXHB} I_{TXHB} \tag{10b}$$

$$\frac{dI_{TXHS}}{dt} = \tau_S \gamma_{BS} I_{HB} - m_{TXHS} I_{TXHS} \tag{11b}$$

$$\frac{dI_{TXHP}}{dt} = \tau_P(\varepsilon_P E_P + \gamma_{BP} I_{HB} + \gamma_{SP} I_{HS}) - m_{TXHP} I_{TXHP} \tag{12b}$$

$$\frac{dR_H}{dt} = m_{HB} g_{HB} I_{HB} + m_{HS} g_{HS} I_{HS} + m_{HP} g_{HP} I_{HP} + m_{TXHB} g_{TXHB} I_{TXHB} + m_{TXHS} g_{TXHS} I_{TXHS} + m_{TXHP} g_{TXHP} I_{TXHP} \tag{13a}$$

$$\frac{dD_H}{dt} = m_{HB}(1 - g_{HB}) I_{HB} + m_{HS}(1 - g_{HS}) I_{HS} + m_{HP}(1 - g_{HP}) I_{HP} + m_{TXHB}(1 - g_{TXHB}) I_{TXHB} + m_{TXHS}(1 - g_{TXHS}) I_{TXHS} + m_{TXHP}(1 - g_{TXHP}) I_{TXHP} \tag{14a}$$

Other health departments in areas with endemic plague have proposed mass antibiotic prophylaxis as a way to combat an outbreak of pneumonic plague.[12] Given the history of plague cases in Madagascar, we considered this recommendation as a potential intervention.[20] The survival probability on oral doxycycline remains 97%, as with treatment.[17 18] We assumed that doxycycline is distributed to susceptible individuals at varying rates (δ_{proph}) from 0 to 10,000 people/day at 1,000 people/day increments. Once individuals move into the doxycycline prophylaxis state, we assumed that they take doxycycline daily for the remainder of the outbreak or until they recover from any potential infection (symptoms present and then recede). Therefore, the number of doses of doxycycline distributed in the model can reach hundreds of millions. If an individual on doxycycline prophylaxis contracts bubonic or pneumonic plague, then that person is not infectious, and the disease does not progress to other forms.

To model doxycycline prophylaxis, we replace equations (7) and (15) with equations (7a) and (15a) and add equations (17-25).

$$\frac{dS_H}{dt} = -\beta_{HB} \frac{S_H}{T_H} F(e^{-\alpha T_R}) - \beta_{HP} \left(\frac{S_H}{T_H}\right) I_{HP} - \delta_{proph} \frac{S_H}{T_H} \tag{7a}$$

$$T_H = S_H + E_{HB} + E_{HP} + I_{HB} + I_{HS} + I_{HP} + I_{TXHB} + I_{TXHS} + I_{TXHP} + R_H + D_H + T_{Hproph} \tag{15a}$$

$$\frac{dS_{Hproph}}{dt} = \delta_{proph} \frac{S_H}{T_H} - \beta_{HBproph} \frac{S_{Hproph}}{T_{Hproph}} F(e^{-\alpha T_R}) - \beta_{HP} \left(\frac{S_{Hproph}}{T_{Hproph}}\right) I_{HP} \tag{17}$$

$$\frac{dE_{Bproph}}{dt} = \beta_{HBproph} \frac{S_{Hproph}}{T_{Hproph}} F(e^{-\alpha T_R}) - \varepsilon_B E_{Bproph} \tag{18}$$

$$\frac{dE_{Pproph}}{dt} = \beta_{HP} \left(\frac{S_{Hproph}}{T_{Hproph}}\right) I_{HP} - \varepsilon_P E_{Pproph} \tag{19}$$

$$\frac{dI_{HBproph}}{dt} = \varepsilon_B E_{Bproph} - m_{TXHB} I_{HBproph} \tag{20}$$

$$\frac{dI_{HPproph}}{dt} = \varepsilon_P E_{Pproph} - m_{TXHP} I_{HPproph} \tag{21}$$

$$\frac{dR_{Hproph}}{dt} = m_{TXHB}g_{TXHB}I_{HBproph} + m_{TXHP}g_{TXHP}I_{HPproph} \quad (22)$$

$$\frac{dD_{Hproph}}{dt} = m_{TXHB}(1 - g_{TXHB})I_{HBproph} + m_{TXHP}(1 - g_{TXHP})I_{HPproph} \quad (23)$$

$$T_{Hproph} = S_{Hproph} + E_{HBproph} + E_{HPproph} + I_{HBproph} + I_{HPproph} + R_{Hproph} + D_{Hproph} \quad (24)$$

$$S_H \geq 0, E_B \geq 0, E_P \geq 0, I_{HB} \geq 0, I_{HS} \geq 0, I_{HP} \geq 0, R_H \geq 0, D_H \geq 0,\ S_{Hproph} \geq 0,$$

$$E_{Bproph} \geq 0, E_{Pproph} \geq 0, I_{HBproph} \geq 0, I_{HPproph} \geq 0, R_{Hproph} \geq 0,\ D_{Hproph} \geq 0 \quad (25)$$

Vector control reduces the incidence of bubonic plague by increasing the mortality rate of fleas.[14] We modeled the mass distribution of malathion to households, with varying coverage levels – 10%-100% in 10% increments – to model discrepancies in compliance with household insecticide use. In every household that is covered by the intervention, fleas will have an increased mortality rate that accounts for the 75% 24-hour mortality probability associated with malathion.

We also varied implementation timing. Rapid interventions start during phase 1 at day 1, 10, 20, 30, 40, 50, or 60 and continue throughout the remainder of the modeled time horizon. Early interventions begin at the start of phase 2 on day 65, 70, 80, or 90. Late interventions begin at the start of phase 3 on day 95, 100, 110, or 120. The different combinations of interventions and their timing and coverage lead to 19,951 different intervention combinations.

Currency, price date and conversion

The salary for community health workers was estimated in 2018 Malagasy Ariary, hospital inpatient price was estimated in 2010 USD, doxycycline price was estimated in 2013 USD, administration cost price was estimated in 2011 USD, and malathion price was estimated in 2004 USD. We converted all costs to 2017 USD using standard annual discounting methods.

Startup Costs

We calculated startup costs bases on intervention timing and coverage level. For intervention timing, we use a linear function to calculate a multiplier $M(t)$ where the cost of an intervention at day 1 is roughly twice as much as that intervention at day 80 and four times as much as that intervention at day 120[21]:

$$M(t) = \frac{-1}{80}t + 2$$

For intervention coverage, we use a linear function to calculate a multiplier $M(c)$ based on the coverage of each intervention type. The multiplier is calculated as a percentage for additional antibiotic treatment and mass distribution of malathion,

$$M(c_{Tx}) = M(c_{Malathion}) = \frac{1}{200}c + 0.65$$

and as a rate of distribution for mass distribution of doxycycline prophylaxis,

$$M\left(c_{Prophylaxis}\right) = \frac{1}{20{,}000}c + 0.65$$

Finally, we adjust the costs associated with each type of intervention by the multipliers to determine the total cost of an intervention:

$$TC = Cost_{Baseline} + M(t)\left[M(c_{Tx})Cost_{Tx} + M\left(c_{Prophylaxis}\right)Cost_{Prophylaxis} + M(c_{Malathion})Cost_{Malathion}\right]$$

Cost Function

The total cost is a combination of the cost of the baseline workers, hospitalizations, additional community health workers (CHWs) to provide additional doxycycline treatment, doxycycline pills for additional treatment, doxycycline pills for mass distribution prophylaxis, administration per person enrolled in mass distribution prophylaxis of doxycycline, malathion per unit, and administration per household covered by mass distribution of malathion. The baseline worker cost includes the cost of employing 1,800 CHWs and 300 doctors throughout the 128 days of the outbreak:

$$Cost_{baseline\ workers} = 2{,}100\ workers * \frac{\$4.58}{day} * 128\ days$$

The hospitalization cost accounts for the infected inpatient hospital costs. We assume 91% of all infected patients sought treatment in a hospital to reflect the case fatality ratio of the outbreak:

$$Cost_{hospitalizaiton} = 0.91 * \frac{\$9.17}{patient \cdot day} * \sum\left(I_{HS} + I_{HP} + I_{TXHB} + I_{TXHS} + I_{TXHP} + I_{HBproph} + I_{HPproph}\right)\ patient \cdot days$$

Additional worker costs include the number of CHWs above baseline that are needed to achieve an improved case fatality ratio when implementing additional doxycycline treatment:

$$Cost_{additional\ workers} = (CFR_{WHO} - CFR_{additional\ treatment}) * n_{workers\ per\ survival\ increase} * \frac{\$4.58}{day} * (128 - day_{intervention})days$$

The cost of doxycycline pills for doxycycline treatment is based on the number of patients taking doxycycline daily:

$$Cost_{doxyTx} = \sum(I_{TXHB} + I_{TXHS} + I_{TXHP})\ patient \cdot days * \frac{\$0.014}{patient \cdot day}$$

Similarly, the cost of doxycycline pills for mass distribution of doxycycline prophylaxis is based on the number of people taking doxycycline daily:

$$Cost_{doxyProph} = \sum \left(S_{Hproph} + E_{HBproph} + E_{HPproph} + I_{HBproph} + I_{HPproph} \right) person \cdot days * \frac{\$0.014}{person \cdot day}$$

The administration cost of mass distribution of doxycycline is based on the enrollment rate of mass distribution of prophylaxis:

$$Cost_{adminProph} = r_{rate\ of\ prophylaxis} \frac{people}{day} * (128 - day_{intervention}) * \frac{\$1.61}{person}$$

The cost of malathion units is based on the number of units delivered to households:

$$Cost_{malathion\ unit} = v_{coverage\ of\ malathion} * \frac{3{,}348{,}794\ people}{4.7\ people\ per\ househould} * \frac{\$4.63}{units \cdot household}$$

The cost of administration for mass distribution of malathion is also based on the number of units delivered to households:

$$Cost_{adminMalathion} = v_{coverage\ of\ malathion} * \frac{3{,}348{,}794\ people}{4.7\ people\ per\ househould} * \frac{\$1.61}{household}$$

Health-Related Quality of Life

The median age in Madagascar is 19.9 years and median life expectancy is 66.6 years.[22] Quality-adjusted life expectancy at birth is 55.0.[23] Given that the quality adjusted life expectancy is less than the median life expectancy, this suggests an average health-related quality of life (HRQoL) of 0.83 per 1 life year. We then assume that each individual in the population is the median age of an individual in Madagascar (~20 years old). The median life expectancy at age 20 is 49.4 years for males and 51.8 years for females.[24] Assuming an equal mix of men and women, the mean life expectancy at age 20 is 50.6 years. After adjusting for the HRQoL of Madagascar and discounting annually, we treated all deaths uniformly as incurring a loss of 23.64 QALYs.

Model of Slower Epidemic

We developed a hypothetical model of a slower epidemic to determine how the intervention decision might change in a future outbreak with different disease dynamics. For bubonic plague, the transmission rate and recovery probability are equal to those of phase 1 of the original model ($\beta_{HB} = 0.2, g_{HB} = 0.9$), while the transmission rate and recovery probability of pneumonic plague are two-thirds and one-third of phase 1 of the original model, respectively ($\beta_{HP} = 0.42, g_{HP} = 0.27$). The slower epidemic model roughly follows the WHO situation reports for bubonic plague, but pneumonic plague cases and overall deaths lag the situation report numbers (Figure S6).

Supplemental Results

Over the 19,951 interventions, the number of cases of pneumonic plague ranged from 113-1,322, the number of cases of bubonic plague ranged from 68-255, and the number of deaths ranged from 18-136.

The number of doses of doxycycline with additional antibiotic treatment varied from 0 - 16. These low figures reflect the already robust antibiotic treatment effort and compounding effects of averted future cases. The number of daily doses of doxycycline prophylaxis reached 148

million while the number of individuals covered reached 1 million and final coverage ranged from 0% - 32% of the overall population. Since vector control was delivered by household as a one-time delivery of a container of malathion, the maximum number of delivered containers was approximately 700,000.

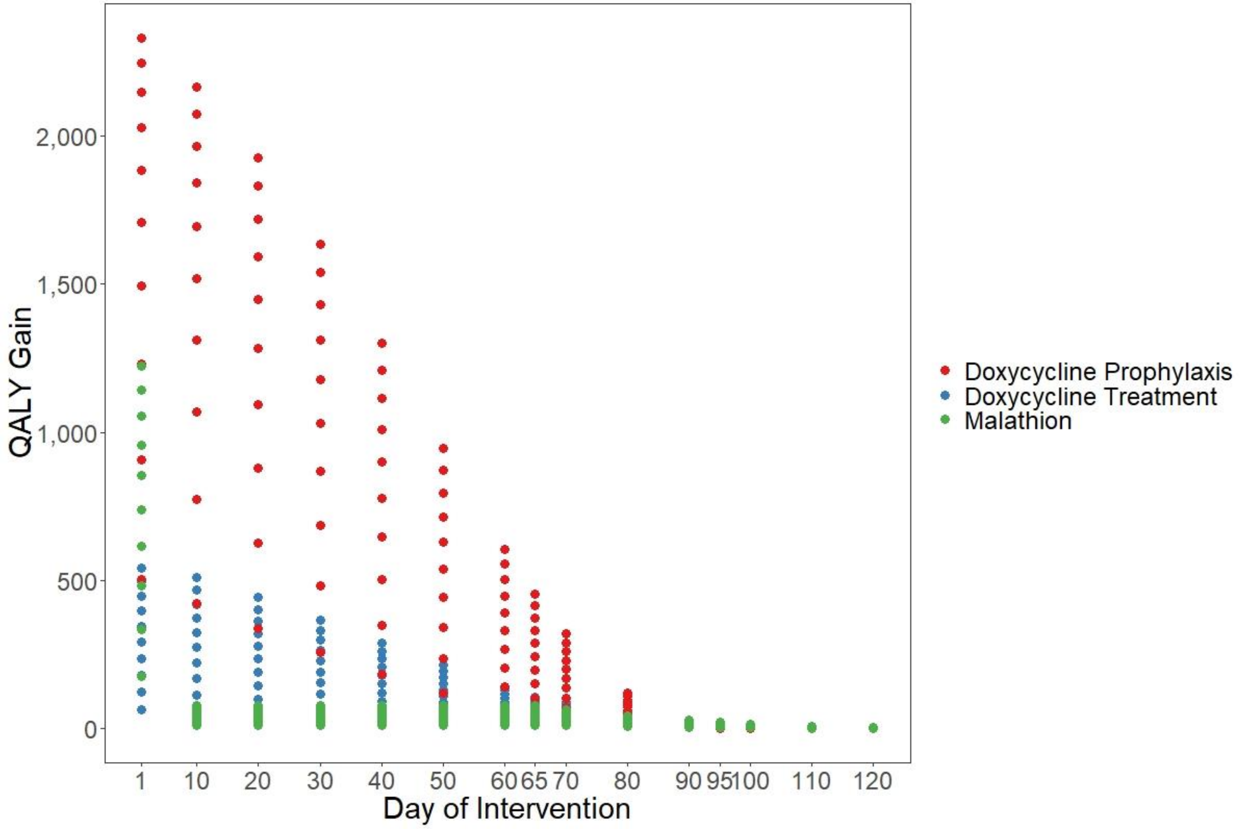

Figure S1. QALYs gained for different interventions as a function of implementation timing. The interventions plotted include only those which have one intervention type

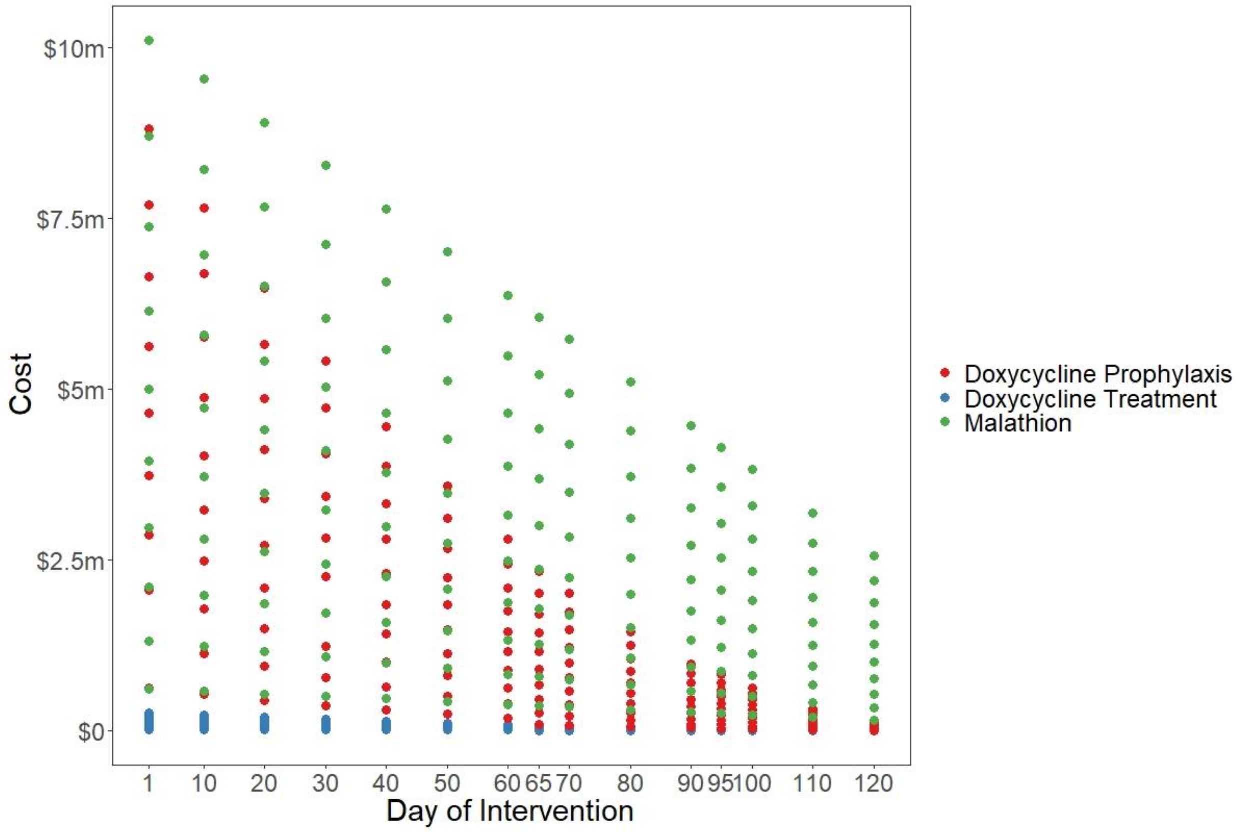

Figure S2. a. Costs of different interventions as a function of implementation timing. The interventions plotted include only those which have one intervention type.

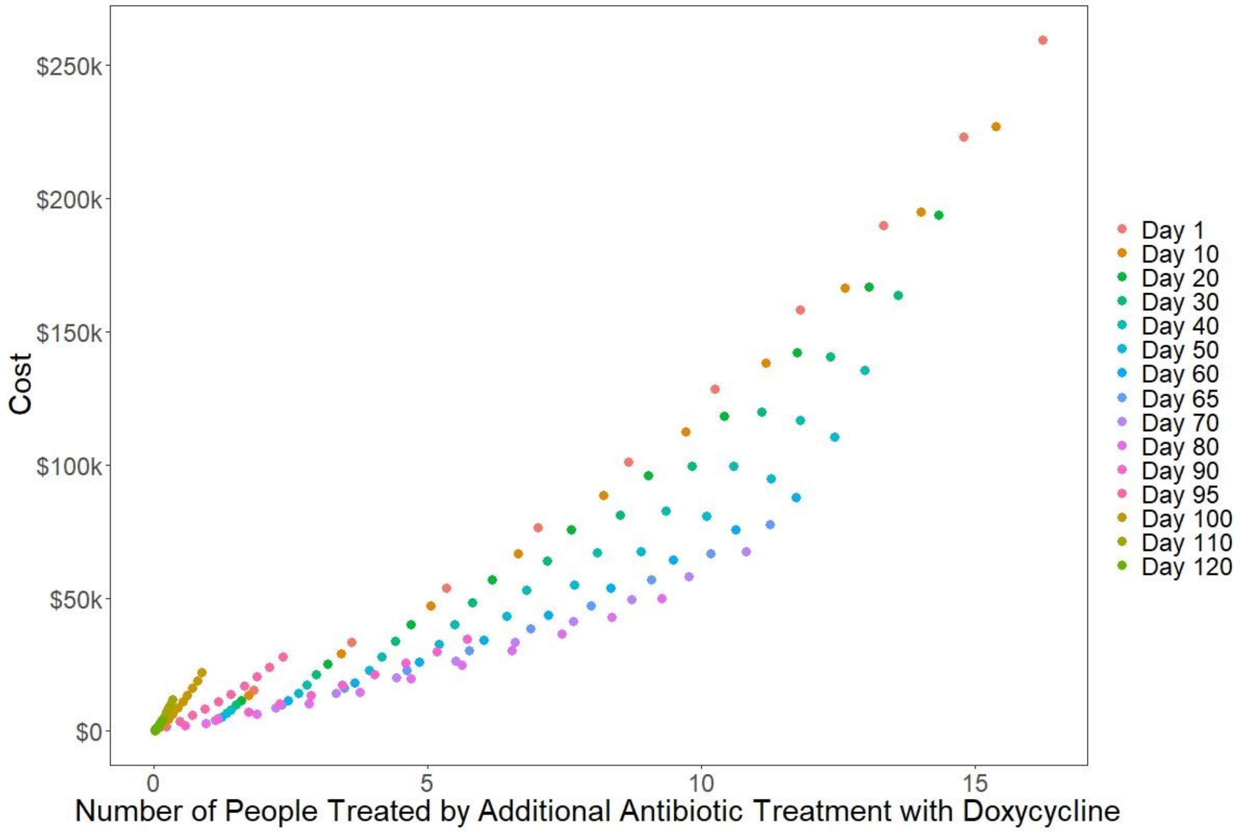

Figure S2. b. Total costs for additional antibiotic treatment with doxycycline as a function of the number of people treated.

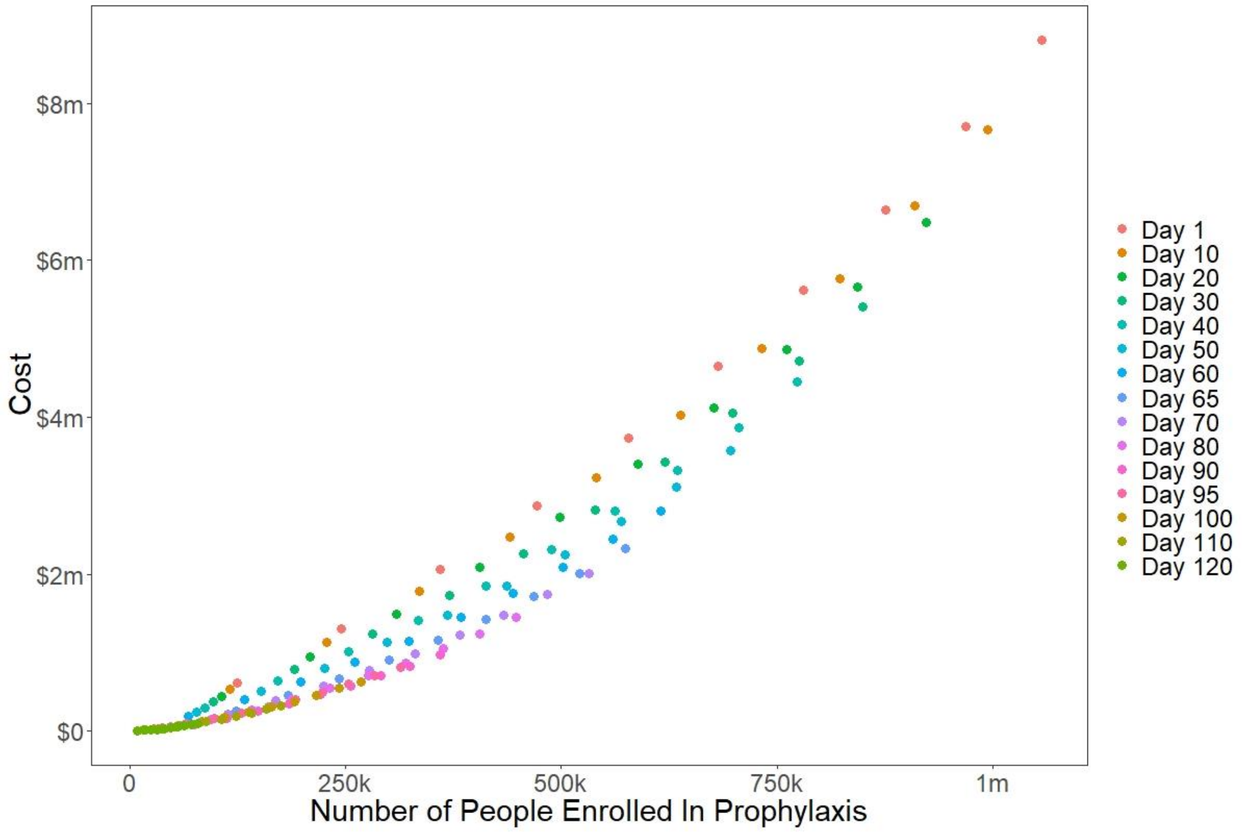

Figure S2. c. Total costs for mass distribution of doxycycline prophylaxis as a function of the number of people enrolled in the mass distribution program.

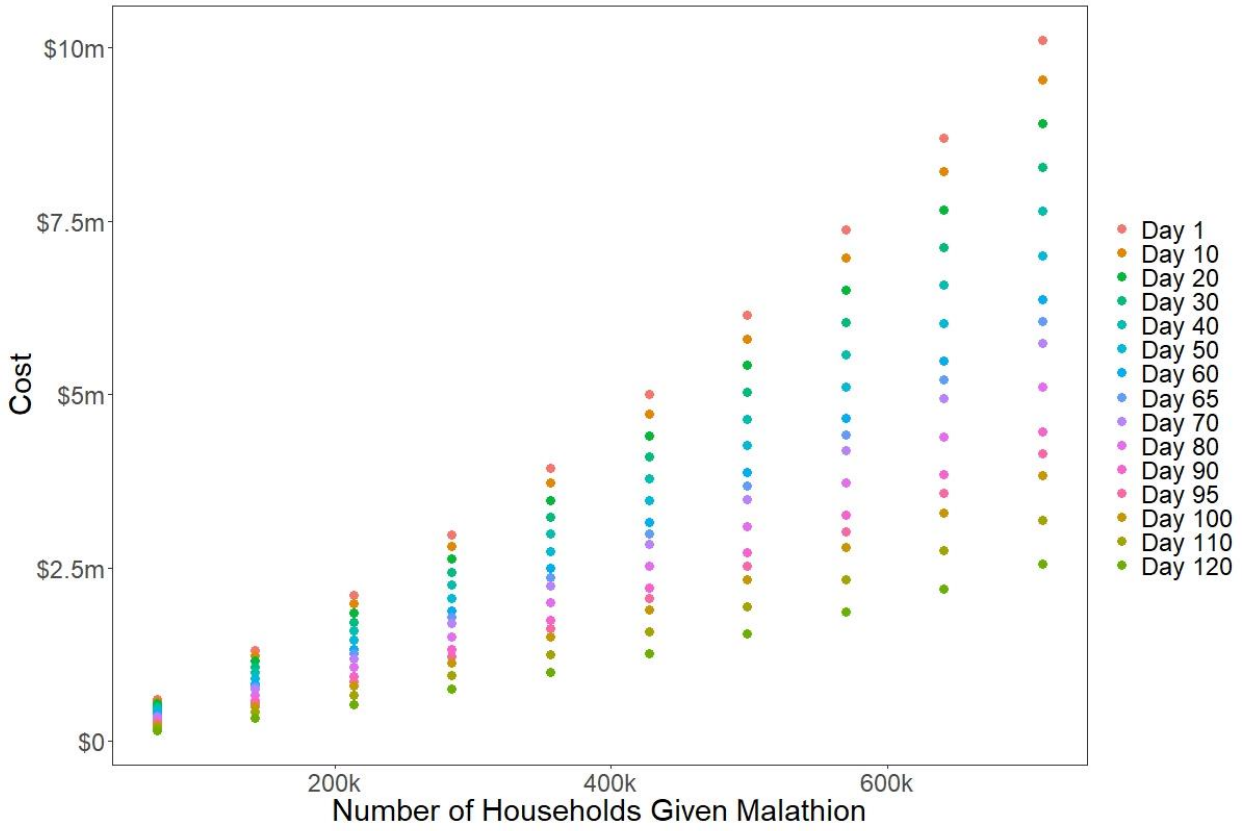

Figure S2. d. Total costs for mass distribution of malathion as a function of the number of households covered.

Figure S2. Intervention costs as a function of implementation timing and coverage

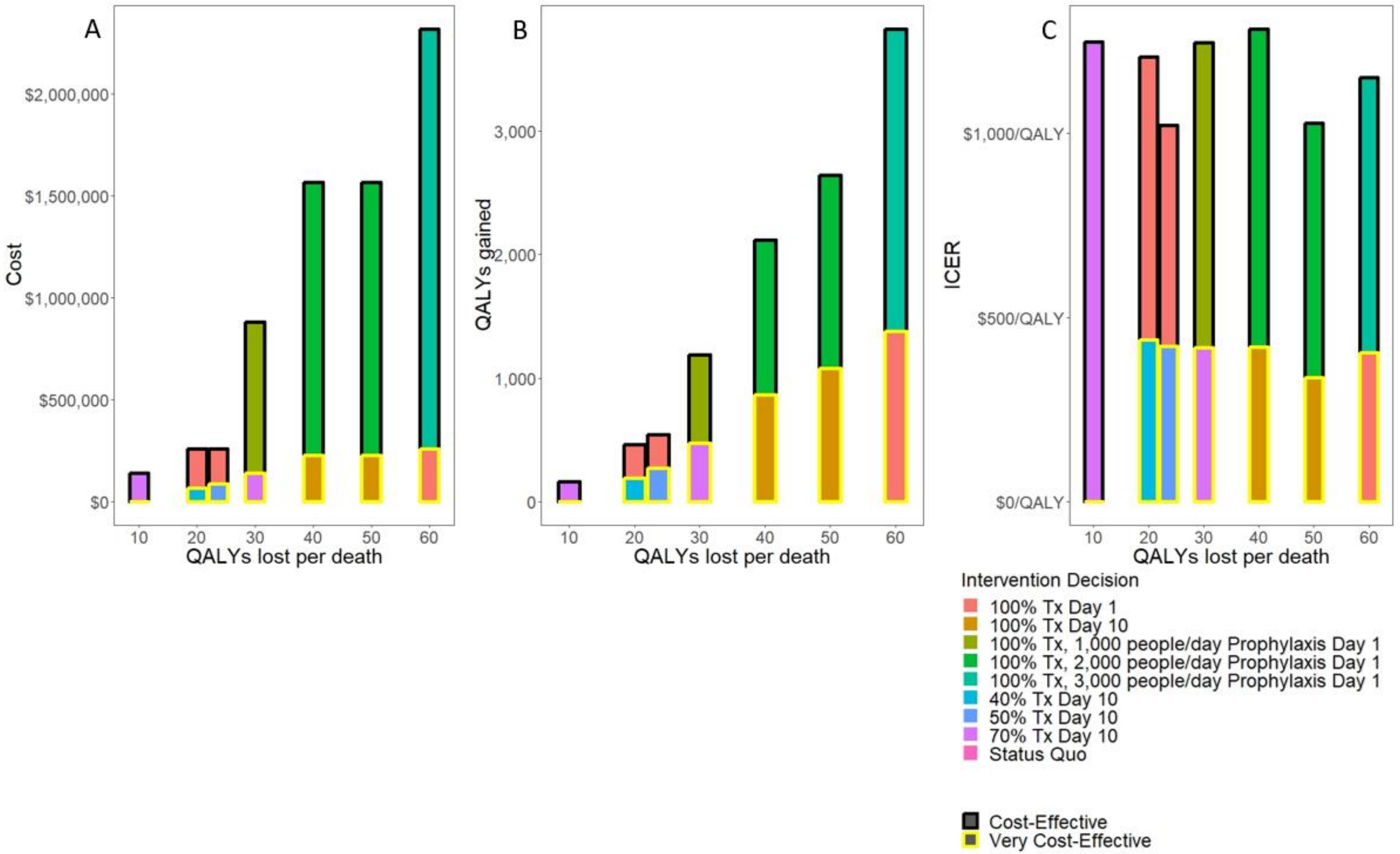

Figure S3. Sensitivity analysis on QALYs lost per death for cost-effective and very cost-effective interventions. Panel A, cost; panel B, QALYs gained; panel C, incremental cost-effectiveness ratio. Panel A shows how the cost differs for the intervention decision at different QALYs lost per death. Outlined in yellow is the total cost and the intervention decision for the very cost-effective threshold (cost/QALY gained less than GDP per capita), and immediately above this outline in black is the total cost and the intervention decision for the cost-

effective threshold (cost/QALY gained less than three times the GDP per capita). Panel B shows the QALYs gained for the same intervention decisions. Panel C shows the incremental cost-effectiveness ratios (ICERs) for the same intervention decisions.

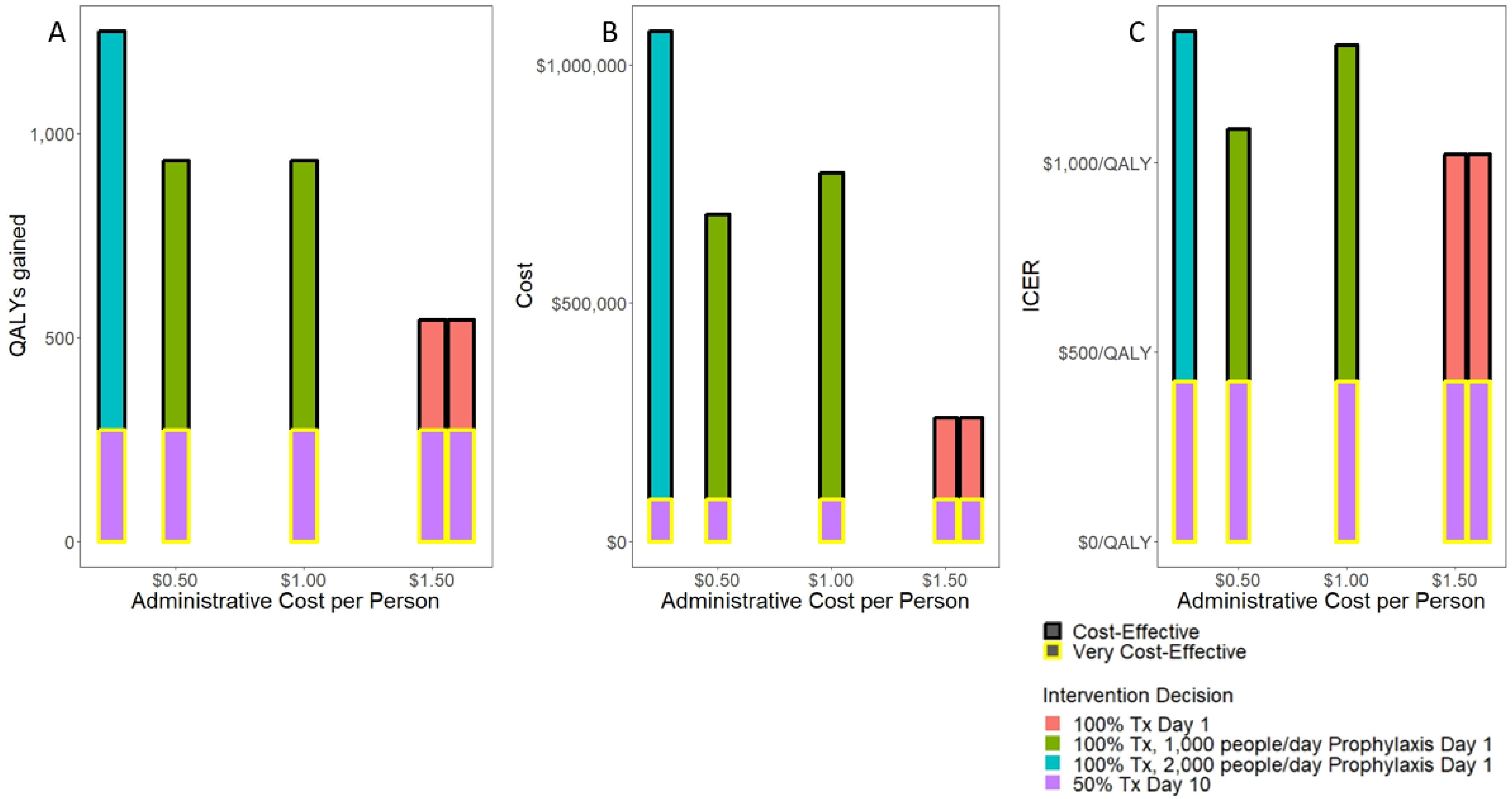

Figure S4. Sensitivity analysis on the administrative cost per person of mass prophylaxis distribution program for cost-effective and very cost-effective interventions. Panel A shows how QALYs gained differ for the intervention decision at different administrative costs per person. Outlined in yellow is the QALYs gained and the intervention decision under the very cost-effective threshold (cost/QALY gained less than GDP per capita), and immediately above this outline in black is the total QALYs gained and the intervention decision under the cost-effective threshold (cost/QALY gained less than three times GDP per capita).

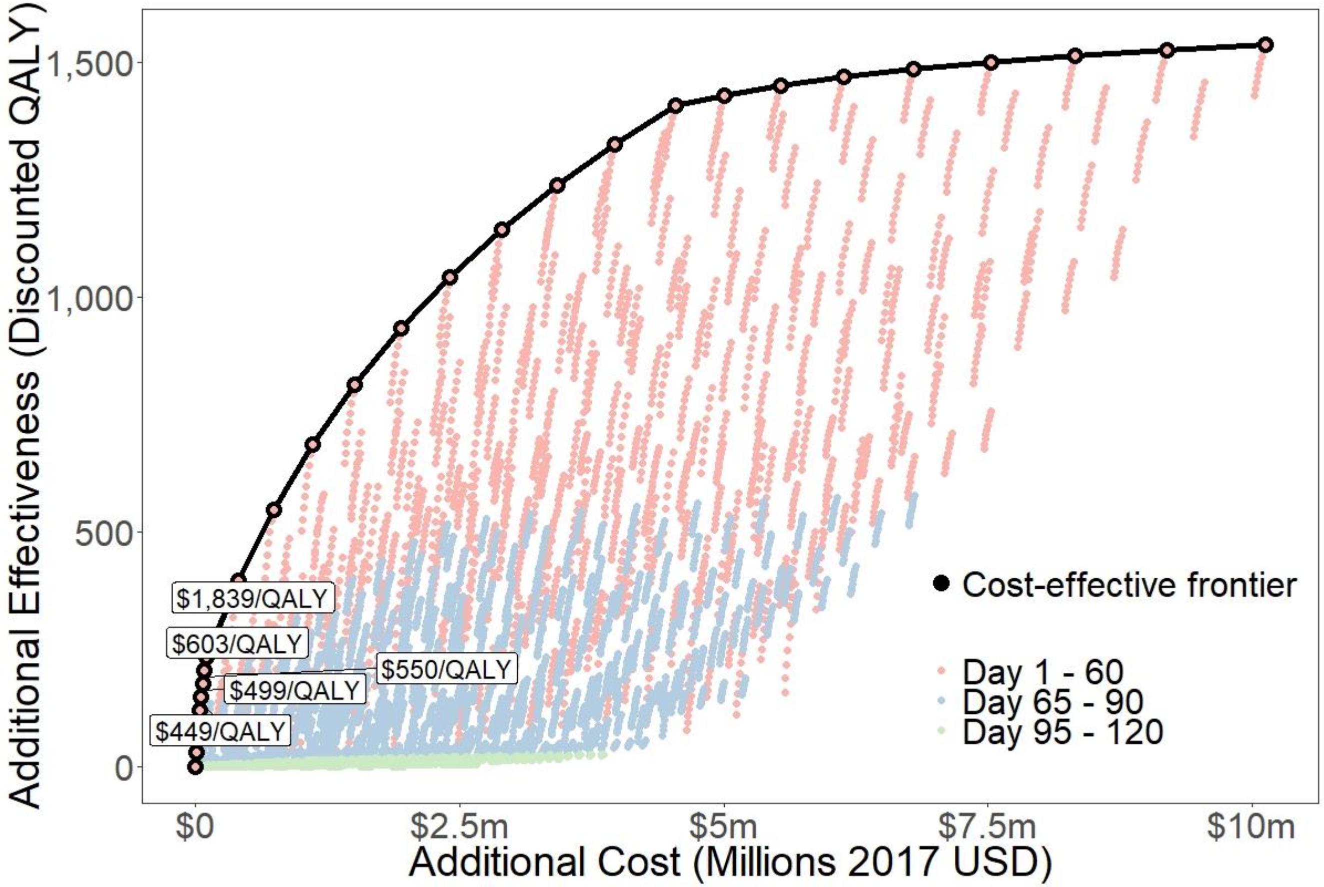

Figure S5. Cost-effectiveness frontier when decision is restricted to interventions day 40 or later where coverage of additional antibiotic treatment is 80% or less and coverage of mass distribution of doxycycline prophylaxis or of malathion is 80% or less.

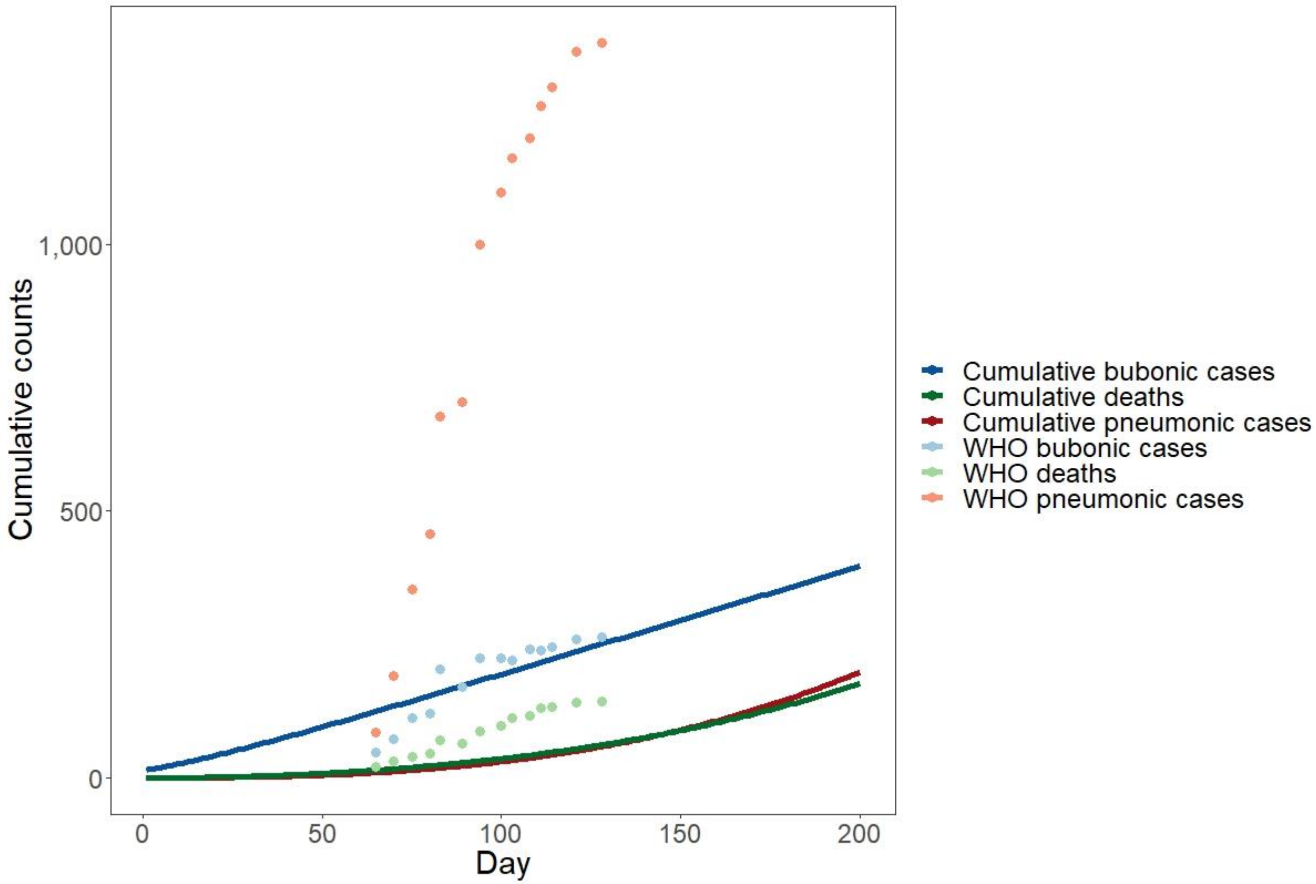

Figure S6. In sensitivity analysis, we developed a model of an outbreak that spreads more slowly than the actual 2017 outbreak. This graph shows cumulative case and death counts for the slower epidemic compared to the WHO situation reports. The bubonic case count follows the WHO case counts for the 2017 outbreak, whereas the number of pneumonic cases and deaths are much lower than the WHO counts.

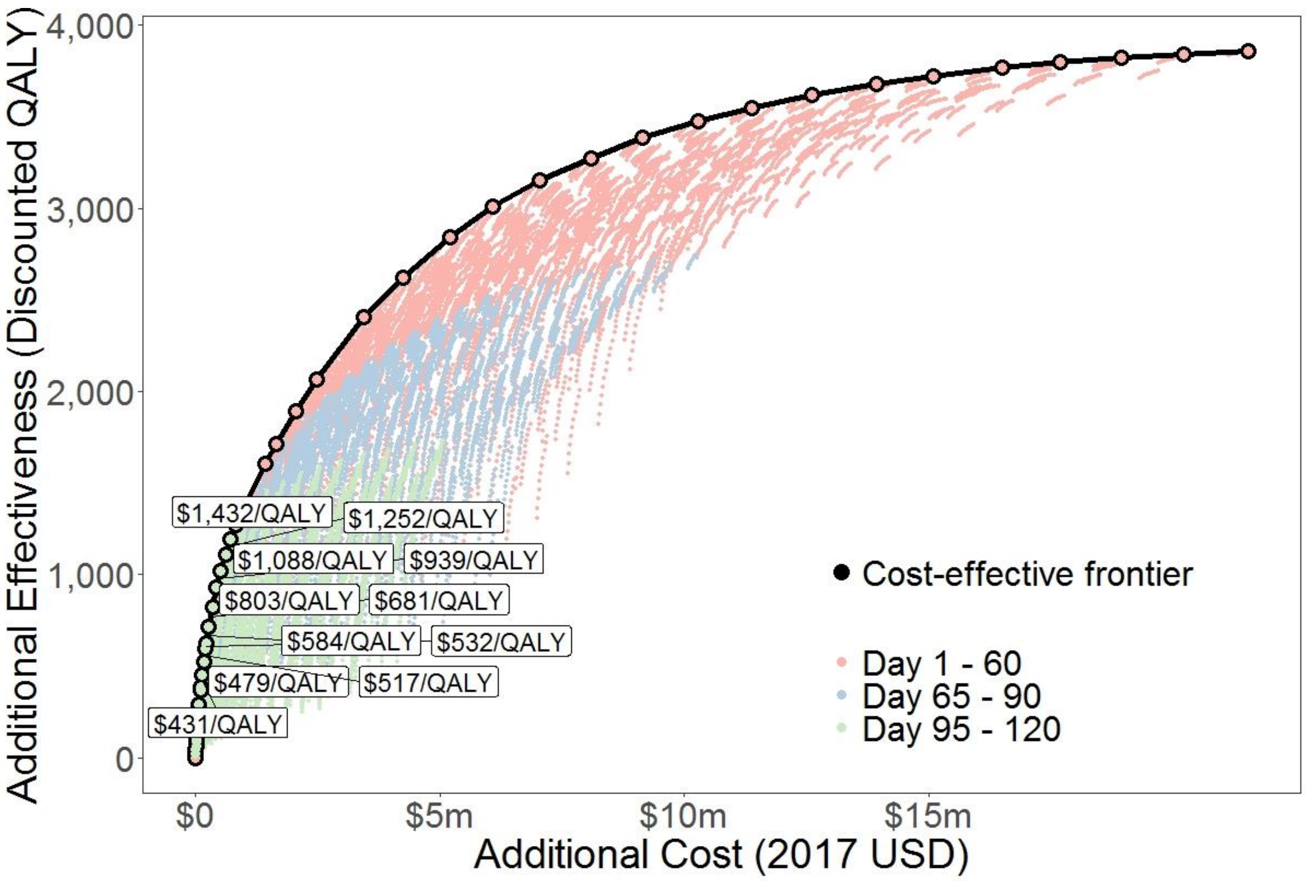

Figure S7. a. Full cost-effectiveness frontier.

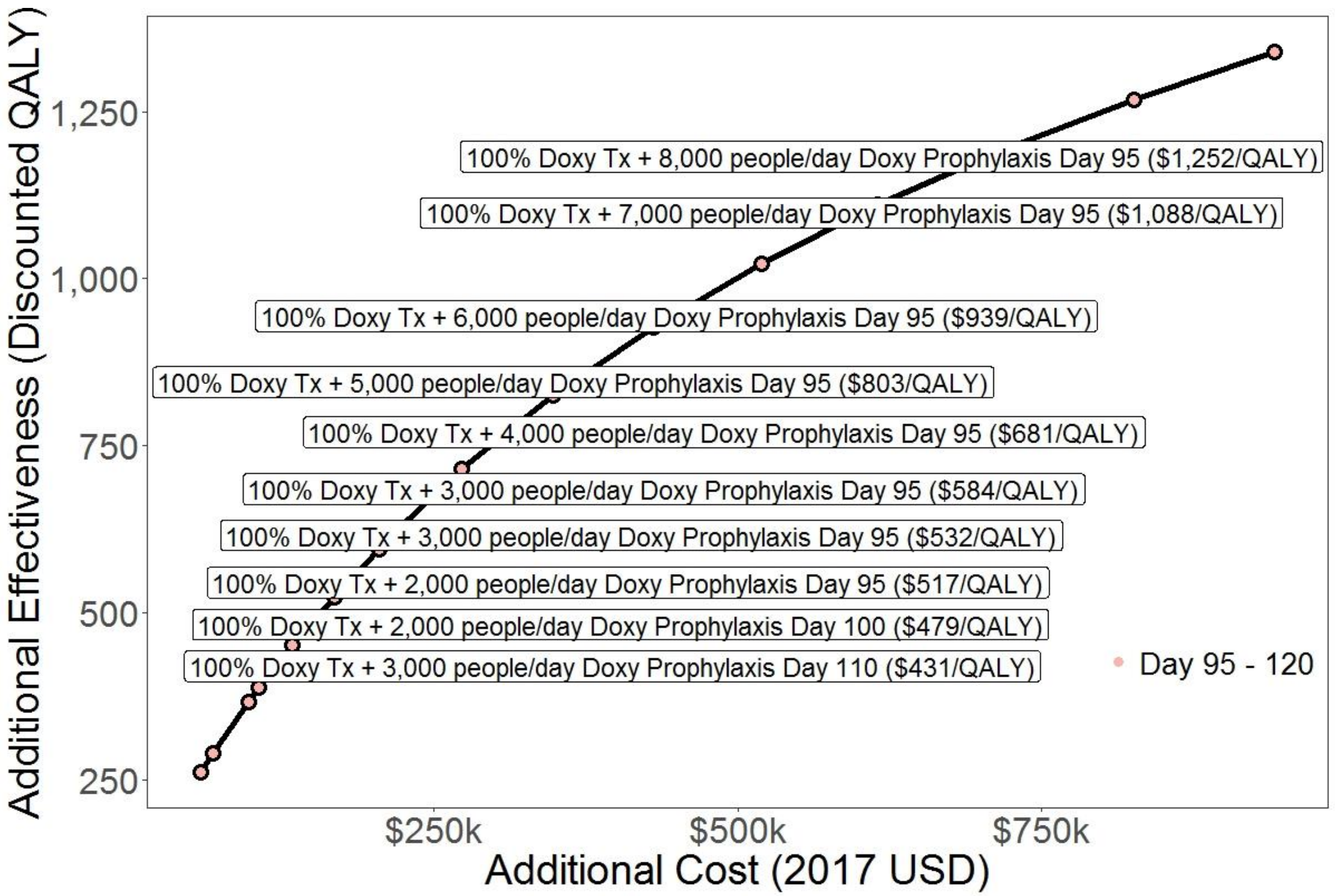

Figure S7. b. Detailed view of cost-effectiveness frontier near the preferred decision.

Figure S7. Cost-effectiveness frontier for slower epidemic.

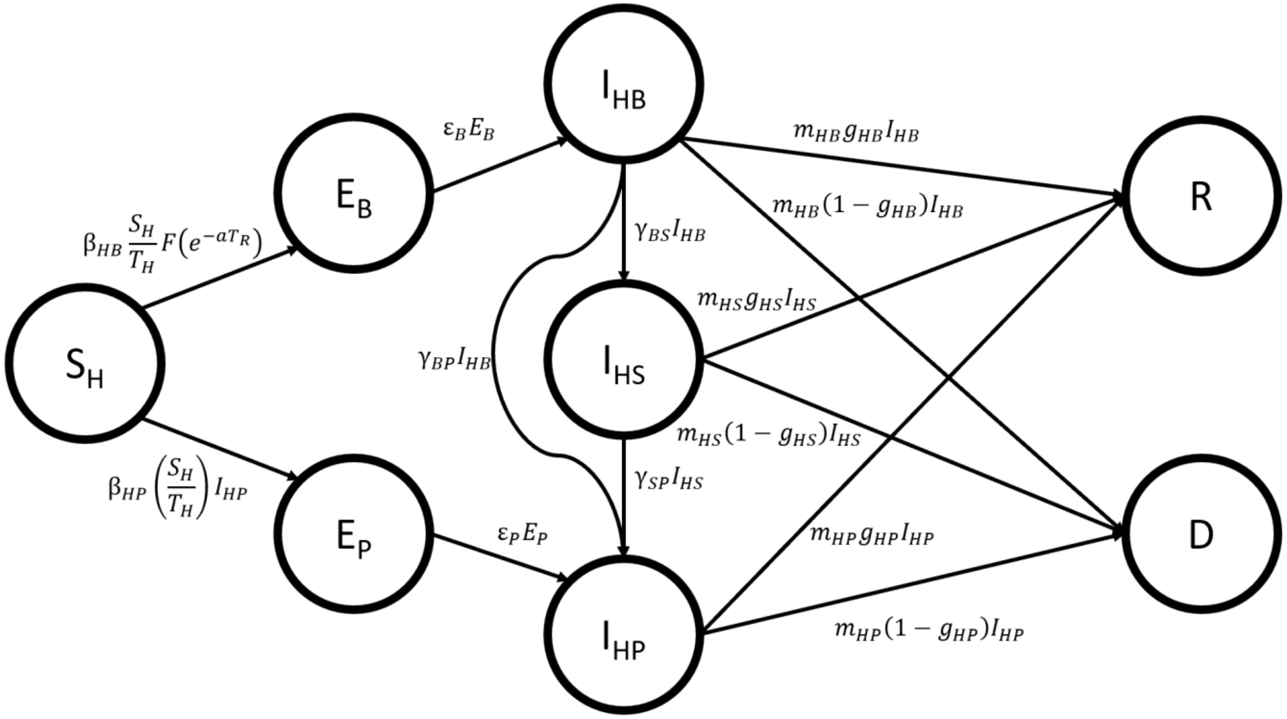

Figure S8. SEIR compartmental model for human plague transmission showing transition parameters.

Table S1. Calibrated Model Projections Compared to WHO Situation Reports, Percent Difference from Adjusted WHO Values.

	**Bubonic Cases**	**Pneumonic Cases**	**Deaths**
Adjusted WHO value	264	1,379	142
Model, day 128	238<br>(-10%)	1,427<br>(+3%)	144<br>(+1%)
Model, end (day 200)	259<br>(-2%)	1,431<br>(+4%)	146<br>(+3%)

Table S2. Costs and Effectiveness of Cost-Effective Interventions for Different Intervention Implementation Days.

Intervention Timing	Cost-Effective Intervention	Total Cost	Total QALYs	Incremental Cost	Incremental QALYs	ICER
Day 1	Additional Antibiotic Treatment (30% Coverage)	$229,800	180.1	$76,630	58.22	$1,316
Day 10	Additional Antibiotic Treatment (100% Coverage)	$542,000	511.0	$54,533	45.07	$1,210
Day 20	Additional Antibiotic Treatment (100% Coverage)	$368,200	441.1	$37,050	39.61	$935
Day 30	Additional Antibiotic Treatment (100% Coverage)	$247,920	364.2	$24,950	33.31	$749
Day 40	Additional Antibiotic Treatment (20% Coverage)	$33,170	61.39	$16,590	30.42	$545
Day 50	Additional Antibiotic Treatment (100% Coverage)	$108,700	213.8	$10,940	20.22	$541
Day 60	Additional Antibiotic Treatment (100% Coverage)	$70,490	141.2	$7,090	13.61	$521
Day 65	Additional Antibiotic Treatment (100% Coverage)	$56,410	106.4	$5,673	10.30	$551
Day 70	Additional Antibiotic Treatment (100% Coverage)	$44,770	87.44	$4,503	8.49	$530
Day 80	Additional Antibiotic Treatment (100% Coverage)	$27,630	49.52	$2,778	4.85	$573
Day 90	Additional Antibiotic Treatment (10% Coverage)	$16,420	15.52	$1,650	1.51	$1,091
Day 95	None	N/A	N/A	N/A	N/A	N/A
Day 100	None	N/A	N/A	N/A	N/A	N/A
Day 110	None	N/A	N/A	N/A	N/A	N/A
Day 120	None	N/A	N/A	N/A	N/A	N/A

QALY = quality-adjusted life year; ICER = incremental cost-effectiveness ratio (cost/QALY gained).

Table S3. Costs and Effectiveness of Interventions on the Cost-Effectiveness Frontier for Different QALY Loss per Death.

QALY Loss per Death	Intervention Timing	Cost-Effective Intervention	Total Cost	Total QALYs	Incremental Cost	Incremental QALYs	ICER
10	Day 10	Additional Antibiotic Treatment (70% Coverage)	$138,300	157.9	$25,880	20.72	$1,249
20	Day 10	Additional Antibiotic Treatment (40% Coverage)	$66,750	187.4	$19,750	44.90	$440
20	Day 1	Additional Antibiotic Treatment (100% Coverage)	$259,400	459.3	$32,380	26.80	$1,208
23.64	Day 10	Additional Antibiotic Treatment (50% Coverage)	$88,550	273.1	$21,800	51.60	$422
23.64	Day 1	Additional Antibiotic Treatment (100% Coverage)	$259,400	542.7	$32,375	31.66	$1,023
30	Day 10	Additional Antibiotic Treatment (70% Coverage)	$138,300	472.0	$25,880	61.97	$418
30	Day 1	Additional Antibiotic Treatment (100% Coverage), Mass Prophylaxis (1,000 people/day)	$879,600	1,186	$620,200	497.5	$1,247
40	Day 10	Additional Antibiotic Treatment (100% Coverage)	$227,000	863.9	$31,950	76.19	$419
40	Day 1	Additional Antibiotic Treatment (100% Coverage), Mass Prophylaxis (2,000 people/day)	$1,567,000	2,116	$687,600	535.8	$1,283
50	Day 10	Additional Antibiotic Treatment (100% Coverage)	$227,000	1,080	$31,950	95.22	$336
50	Day 1	Additional Antibiotic Treatment (100% Coverage), Mass Prophylaxis (2,000 people/day)	$1,567,000	2,645	$687,600	669.6	$1,027
60	Day 1	Additional Antibiotic Treatment (100% Coverage)	$259,400	1,376	$32,380	80.26	$403
60	Day 1	Additional Antibiotic Treatment (100% Coverage), Mass Prophylaxis (3,000 people/day)	$2,318,000	3,825	$751,100	652.5	$1,151

QALY = quality-adjusted life year; ICER = incremental cost-effectiveness ratio (cost/QALY gained).

Table S4. Costs and Effectiveness of Interventions on the Cost-Effectiveness Frontier for Different Administrative Cost per Person of Mass Distribution.

Administrative Cost per Person	Intervention Timing	Cost-Effective Intervention	Total Cost	Total QALYs	Incremental Cost	Incremental QALYs	ICER
$0.25	Day 10	Additional Antibiotic Treatment (50% Coverage)	$88,550	273.1	$21,800	51.60	$422
$0.25	Day 1	Additional Antibiotic Treatment (100% Coverage), Mass Prophylaxis (2,000 people/day)	$1,701,000	1,252	$427,300	317.0	$1,348
$0.50	Day 10	Additional Antibiotic Treatment (50% Coverage)	$88,550	273.1	$21,800	51.60	$422
$0.50	Day 1	Additional Antibiotic Treatment (100% Coverage), Mass Prophylaxis (1,000 people/day)	$687,000	934.9	$427,600	392.3	$1,090
$1.00	Day 10	Additional Antibiotic Treatment (50% Coverage)	$88,550	273.1	$21,800	51.60	$422
$1.00	Day 1	Additional Antibiotic Treatment (100% Coverage), Mass Prophylaxis (1,000 people/day)	$773,600	934.9	$514,300	392.3	$1,311
$1.50	Day 10	Additional Antibiotic Treatment (50% Coverage)	$88,550	273.1	$21,800	51.60	$422
$1.50	Day 1	Additional Antibiotic Treatment (100% Coverage)	$259,400	542.7	$32,380	31.66	$1,023
$1.61	Day 10	Additional Antibiotic Treatment (50% Coverage)	$88,550	273.1	$21,800	51.60	$422
$1.61	Day 1	Additional Antibiotic Treatment (100% Coverage)	$259,400	542.7	$32,380	31.66	$1,023

QALY = quality-adjusted life year; ICER = incremental cost-effectiveness ratio.

Table S5. Costs and Effectiveness of Interventions on the Cost-Effectiveness Frontier for a Slower Epidemic.

Intervention Timing	Doxycycline Treatment Additional Coverage	Doxycycline Prophylaxis Distribution Rate, People/Day (Final Coverage as % of Total Population)	Malathion Distribution Coverage	Total Cost	Total QALYs	Incremental Cost	Incremental QALYs	ICER
N/A	0%	0 (0%)	0%	$0	0	N/A	N/A	N/A
Day 120	10%	0 (0%)	0%	$2,542	22.12	$2,542	22.12	$115

Intervention Timing	Doxycycline Treatment Additional Coverage	Doxycycline Prophylaxis Distribution Rate, People/Day (Final Coverage as % of Total Population)	Malathion Distribution Coverage	Total Cost	Total QALYs	Incremental Cost	Incremental QALYs	ICER
Day 120	20%	0 (0%)	0%	$5,450	43.79	$2,909	21.67	$134
Day 120	30%	0 (0%)	0%	$8,726	65.07	$3,276	21.28	$154
Day 120	40%	0 (0%)	0%	$12,370	85.97	$3,643	20.90	$174
Day 120	50%	0 (0%)	0%	$16,380	106.5	$4,010	20.53	$195
Day 120	60%	0 (0%)	0%	$20,760	126.7	$4,378	20.18	$217
Day 120	70%	0 (0%)	0%	$25,500	146.5	$4,745	19.81	$239
Day 120	70%	1,000 (0.2%)	0%	$33,050	176.8	$7,547	30.34	$249
Day 120	80%	1,000 (0.2%)	0%	$38,160	195.9	$5,112	19.03	$269
Day 120	90%	1,000 (0.2%)	0%	$43,440	214.6	$5,278	18.72	$282
Day 120	90%	2,000 (0.5%)	0%	$52,060	243.5	$8,621	28.91	$298
Day 120	100%	2,000 (0.5%)	0%	$57,900	261.5	$5,838	17.99	$324
Day 120	100%	3,000 (0.7%)	0%	$67,590	289.5	$9,689	28.02	$346
Day 110	90%	2,000 (1.1%)	0%	$97,140	366.9	$29,560	77.36	$382
Day 110	100%	2,000 (1.1%)	0%	$105,400	387.5	$8,215	20.62	$398
Day 110	100%	3,000 (1.6%)	0%	$133,200	452.1	$27,830	64.61	$431
Day 100	100%	2,000 (1.7%)	0%	$167,500	523.7	$34,320	71.64	$479
Day 95	100%	2,000 (2.0%)	0%	$204,300	594.9	$36,810	71.15	$517
Day 100	100%	3,000 (2.5%)	0%	$220,500	625.3	$16,200	30.44	$532
Day 95	100%	3,000 (2.9%)	0%	$272,700	714.6	$52,140	89.33	$584
Day 95	100%	4,000 (3.9%)	0%	$348,000	825.3	$75,360	110.7	$681
Day 95	100%	5,000 (4.8%)	0%	$430,300	927.8	$82,280	102.4	$803
Day 95	100%	6,000 (5.7%)	0%	$519,400	1,023	$89,100	94.92	$939
Day 95	100%	7,000 (6.7%)	0%	$615,200	1,111	$95,810	88.05	$1,088
Day 95	100%	8,000 (7.6%)	0%	$717,600	1,193	$102,400	81.79	$1,252
Day 95	100%	9,000 (8.5%)	0%	$826,600	1,269	$108,900	76.06	$1,432
Day 95	100%	10,000 (9.4%)	0%	$941,900	1,339	$115,400	70.83	$1,629
Day 30	100%	2,000 (9.7%)	0%	$1,421,000	1,603	$479,300	263.2	$1,821
Day 20	100%	2,000 (10%)	0%	$1,651,000	1,715	$229,600	112.0	$2,051
Day 30	100%	3,000 (14%)	0%	$2,063,000	1,893	$412,200	178.3	$2,312
Day 10	100%	2,000 (11%)	10%	$2,475,000	2,063	$412,200	170.1	$2,424
Day 1	100%	2,000 (11%)	20%	$3,451,000	2,404	$975,600	341.3	$2,858
Day 1	100%	2,000 (11%)	30%	$4,238,000	2,622	$787,100	217.8	$3,613
Day 1	100%	3,000 (16%)	30%	$5,211,000	2,841	$973,300	219.0	$4,444
Day 1	100%	3,000 (16%)	40%	$6,088,000	3,008	$876,700	116.8	$5,257
Day 1	100%	3,000 (16%)	50%	$7,054,000	3,152	$965,900	144.3	$6,695

Intervention Timing	Doxycycline Treatment Additional Coverage	Doxycycline Prophylaxis Distribution Rate, People/Day (Final Coverage as % of Total Population)	Malathion Distribution Coverage	Total Cost	Total QALYs	Incremental Cost	Incremental QALYs	ICER
Day 1	100%	4,000 (21%)	50%	$8,091,000	3,274	$1,037,000	122.1	$8,494
Day 1	100%	4,000 (21%)	60%	$9,146,000	3,384	$1,055,000	109.9	$9,605
Day 1	100%	4,000 (21%)	70%	$10,290,000	3,477	$1,144,000	93.00	$12,304
Day 1	100%	5,000 (26%)	70%	$11,390,000	3,548	$1,095,000	70.73	$15,488
Day 1	100%	5,000 (26%)	80%	$12,620,000	3,619	$1,233,000	70.89	$17,400
Day 1	100%	5,000 (26%)	90%	$13,940,000	3,678	$1,322,000	59.54	$22,200
Day 1	100%	6,000 (30%)	90%	$15,090,000	3,721	$1,149,000	42.88	$26,790
Day 1	100%	6,000 (30%)	100%	$16,500,000	3,767	$1,411,000	45.94	$30,710
Day 1	100%	7,000 (34%)	100%	$17,700,000	3,797	$1,197,000	30.10	$39,780
Day 1	100%	8,000 (38%)	100%	$18,940,000	3,821	$1,242,000	24.11	$51,500
Day 1	100%	9,000 (41%)	100%	$20,220,000	3,841	$1,282,000	19.81	$64,710
Day 1	100%	10,000 (45%)	100%	$21,540,000	3,858	$1,319,000	16.64	$79,240

QALY = quality-adjusted life year; ICER = incremental cost-effectiveness ratio (cost/QALY gained).

Tropical Medicine and Infectious Disease

MDPI

CHEERS (Consolidated Health Economic Evaluation Reporting System) Checklist.

Section/item	Item No	Recommendation	Section Where Reported
**Title and abstract**			
Title	1	Identify the study as an economic evaluation or use more specific terms such as "cost-effectiveness analysis", and describe the interventions compared.	Title (page 1)
Abstract	2	Provide a structured summary of objectives, perspective, setting, methods (including study design and inputs), results (including base case and uncertainty analyses), and conclusions.	Abstract (page 2)
**Introduction**			
Background and objectives	3	Provide an explicit statement of the broader context for the study.	Introduction, ¶1-4 (page 4)
		Present the study question and its relevance for health policy or practice decisions.	Introduction, ¶5-7 (page 4-5)
**Methods**			
Target population and subgroups	4	Describe characteristics of the base case population and subgroups analysed, including why they were chosen.	Methods: Model (page 5-6)
Setting and location	5	State relevant aspects of the system(s) in which the decision(s) need(s) to be made.	Methods: Interventions (page 6-8)
Study perspective	6	Describe the perspective of the study and relate this to the costs being evaluated.	Abstract (page 2), Methods: Cost-effectiveness analysis (page 9-10)
Comparators	7	Describe the interventions or strategies being compared and state why they were chosen.	Methods: Interventions (page 6-8)
Time horizon	8	State the time horizon(s) over which costs and consequences are being evaluated and say why appropriate.	Methods: Model (page 5-6)
Discount rate	9	Report the choice of discount rate(s) used for costs and outcomes and say why appropriate.	Methods: Cost-effectiveness Analysis (page 9-10)
Choice of health outcomes	10	Describe what outcomes were used as the measure(s) of benefit in the evaluation and their relevance for the type of analysis performed.	Methods: Cost-effectiveness Analysis (page 9-10)
Measurement of effectiveness	11a	*Single study-based estimates:* Describe fully the design features of the single effectiveness study and why the single study was a sufficient source of clinical effectiveness data.	Methods: Interventions (page 6-8), Supplement: Model Details (page S1-S12), Supplement: Health-related Quality of Life (page S12)
	11b	*Synthesis-based estimates*: Describe fully the methods used for identification of included studies and synthesis of clinical effectiveness data.	Not applicable
Measurement and valuation of preference based outcomes	12	If applicable, describe the population and methods used to elicit preferences for outcomes.	Methods: Cost-effectiveness Analysis (page 9-10)
Estimating resources and costs	13a	*Single study-based economic evaluation:* Describe approaches used to estimate resource use associated with the alternative interventions. Describe primary or secondary research methods for valuing each resource item in terms of its unit cost. Describe any adjustments made to approximate to opportunity costs.	Not applicable

Section/item	Item No	Recommendation	Section Where Reported
	13b	*Model-based economic evaluation:* Describe approaches and data sources used to estimate resource use associated with model health states. Describe primary or secondary research methods for valuing each resource item in terms of its unit cost. Describe any adjustments made to approximate to opportunity costs.	Methods: Model (page 5-6), Methods: Interventions (page 6-8), Methods: Cost-effectiveness Analysis (page 9-10), Supplement: Model Details (page S1-S12)
Currency, price date, and conversion	14	Report the dates of the estimated resource quantities and unit costs. Describe methods for adjusting estimated unit costs to the year of reported costs if necessary. Describe methods for converting costs into a common currency base and the exchange rate.	Methods: Cost-effectiveness Analysis (page 9-10), Supplement: Model Details (page S1-S12)
Choice of model	15	Describe and give reasons for the specific type of decision-analytical model used. Providing a figure to show model structure is strongly recommended.	Methods: Model (page 5-6), Supplement: Model Details (page S1-S12)
Assumptions	16	Describe all structural or other assumptions underpinning the decision-analytical model.	Methods: Model (page 5-6), Methods: Interventions (page 6-8), Methods: Cost-effectiveness Analysis (page 9-10), Supplement: Model Details (page S1-S12), Supplement: Health-related Quality of Life (page S12)
Analytical methods	17	Describe all analytical methods supporting the evaluation. This could include methods for dealing with skewed, missing, or censored data; extrapolation methods; methods for pooling data; approaches to validate or make adjustments (such as half cycle corrections) to a model; and methods for handling population heterogeneity and uncertainty.	Methods: Cost-effectiveness Analysis (page 9-10), Supplement: Model details (page S1-S12)
		**Results**	
Study parameters	18	Report the values, ranges, references, and, if used, probability distributions for all parameters. Report reasons or sources for distributions used to represent uncertainty where appropriate. Providing a table to show the input values is strongly recommended.	Tables 1, 2 (page 26-28)
Incremental costs and outcomes	19	For each intervention, report mean values for the main categories of estimated costs and outcomes of interest, as well as mean differences between the comparator groups. If applicable, report incremental cost-effectiveness ratios.	Tables 3 (page 29-30), S2 (page S28), S3 (page S29-S30), S4 (page S31), S5 (page S32-S34)
Characterising uncertainty	20a	*Single study-based economic evaluation:* Describe the effects of sampling uncertainty for the estimated incremental cost and incremental effectiveness parameters, together with the impact of methodological assumptions (such as discount rate, study perspective).	Not applicable
	20b	*Model-based economic evaluation:* Describe the effects on the results of uncertainty for all input	Results: Sensitivity Analysis (page 13-14), Supplement:

Section/item	Item No	Recommendation	Section Where Reported
		parameters, and uncertainty related to the structure of the model and assumptions.	Supplemental Results (page S13)
Characterising heterogeneity	21	If applicable, report differences in costs, outcomes, or cost-effectiveness that can be explained by variations between subgroups of patients with different baseline characteristics or other observed variability in effects that are not reducible by more information.	Not applicable
**Discussion**			
Study findings, limitations, generalisability, and current knowledge	22	Summarise key study findings and describe how they support the conclusions reached. Discuss limitations and the generalisability of the findings and how the findings fit with current knowledge.	Discussion (page 14-18)
**Other**			
Source of funding	23	Describe how the study was funded and the role of the funder in the identification, design, conduct, and reporting of the analysis. Describe other non-monetary sources of support.	Information provided via the submission system
Conflicts of interest	24	Describe any potential for conflict of interest of study contributors in accordance with journal policy. In the absence of a journal policy, we recommend authors comply with International Committee of Medical Journal Editors recommendations.	Information provided via the submission system

References

1. Keeling MJ, Gilligan CA. Bubonic plague: a metapopulation model of a zoonosis. *Proc Biol Sci* 2000;267(1458):2219-30.
2. Keeling MJ, Gilligan CA. Metapopulation dynamics of bubonic plague. *Nature* 2000;407(6806):903-6.
3. World Health Organization. Plague outbreak Madagascar, External situation report 14. 2017 [Available from: https://www.afro.who.int/health-topics/plague/plague-outbreak-situation-reports accessed Jun 20 2019.
4. Buckle AP, Smith RH, editors. *Rodent Pests and Their Control*. Wallingford, UK: CAB International, 1994.
5. Hirst LF. Plague. British Encyclopedia of Medical Practice, Volume 9: Butterworth and Co 1938:675-98.
6. Wheeler CM, Douglas JR. Sylvatic plague studies: V. The determination of vector efficiency. *J Infect Dis* 1945;77(1):1-12.
7. Macchiavello A. Reservoirs and vectors of plague. *J Trop Med Hyg* 1954;57(12):294-8; concl.
8. Hinnebusch BJ, Gage KL, Schwan TG. Estimation of vector infectivity rates for plague by means of a standard curve-based competitive polymerase chain reaction method to quantify *Yersinia pestis* in fleas. *Am J Trop Med Hyg* 1998;58(5):562-9.
9. Gani R, Leach S. Epidemiological determinants for modeling pneumonic plague outbreaks. *Emerg Infect Dis* 2004;10(4):608-14.
10. Tsuzuki S, Lee H, Miura F, et al. Dynamics of the pneumonic plague epidemic in Madagascar, August to October 2017. *Euro Surveill* 2017;22(46)
11. Centers for Disease Control and Prevention. Recommended antibiotic treatment for plague 2015 [Available from: https://www.cdc.gov/plague/resources/Recommended-antibiotics-for-plague_revision-Aug-2015_Final-(00000002).pdf accessed Jun 20 2019.
12. New Mexico Department of Health. Mass post exposure prophylaxis protocol: New Mexico Department of Health; 2013 [Available from: https://nmhealth.org/publication/view/policy/2964/ accessed Jun 20 2019.
13. Oklahoma State Department of Health. Plague prophylaxis (bioterrorism) 2018 [Available from: https://www.ok.gov/health2/documents/Plague%20Prophylaxis.pdf accessed Jun 20 2019.
14. World Health Organization. Interregional meeting on prevention and control of plague. Epidemic and Pandemic Alert and Response: World Health Organization, 2006.
15. Brouillard JE, Terriff CM, Tofen A, et al. Antibiotic selection and resistance issues with fluoroquinolones and doxycycline against bioterrorism agents. *Pharmacotherapy* 2006;26(1):3-14.
16. Inglesby TV, Dennis DT, Henderson DA, et al. Plague as a biological weapon. *JAMA* 2000;283(17):2281-90. doi: 10.1001/jama.283.17.2281
17. Mwengee W, Butler T, Mgema S, et al. Treatment of plague with gentamicin or doxycycline in a randomized clinical trial in Tanzania. *Clin Infect Dis* 2006;42(5):614-21. [published Online First: 2006/01/25]
18. Narayanan N, Lacy CR, Cruz JE, et al. Disaster preparedness: biological threats and treatment options. *Pharmacotherapy* 2018;38(2):217-34. [published Online First: 2018/01/05]
19. Hinckley AF, Biggerstaff BJ, Griffith KS, et al. Transmission dynamics of primary pneumonic plague in the USA. *Epidemiol Infect* 2012;140(3):554-60. [published Online First: 2011/07/07]
20. Hull HF, Montes JM, Mann JM. Septicemic plague in New Mexico. *J Infect Dis* 1987;155(1):113-8.
21. Johns B, Baltussen R, Hutubessy R. Programme costs in the economic evaluation of health interventions. *Cost Eff Resour Alloc* 2003;1(1):1. [published Online First: 2003/05/30]
22. Central Intelligence Agency. The World Factbook: Madagascar 2018 [Available from: https://www.cia.gov/library/publications/the-world-factbook/geos/ma.html accessed May 3 2018.
23. World Health Organization. Madagascar: WHO statistical profile 2015 [Available from: https://www.who.int/gho/countries/mdg/data/en/2019.
24. World Health Organization. Life tables by country: Madagascar 2016 [Available from: http://apps.who.int/gho/data/?theme=main&vid=609702019.
